# The Cannabinoid Receptor CB1 Interacts with the WAVE1 Complex and Plays a Role in Actin Dynamics and Structural Plasticity in Neurons

**DOI:** 10.1371/journal.pbio.1002286

**Published:** 2015-10-23

**Authors:** Christian Njoo, Nitin Agarwal, Beat Lutz, Rohini Kuner

**Affiliations:** 1 Pharmacology Institute, Medical Faculty Heidelberg, Heidelberg University, Heidelberg, Germany; 2 Institute of Physiological Chemistry, University Medical Center of the Johannes Gutenberg University Mainz, Mainz, Germany; Mount Sinai School of Medicine, UNITED STATES

## Abstract

The molecular composition of the cannabinoid type 1 (CB1) receptor complex beyond the classical G-protein signaling components is not known. Using proteomics on mouse cortex in vivo, we pulled down proteins interacting with CB1 in neurons and show that the CB1 receptor assembles with multiple members of the WAVE1 complex and the RhoGTPase Rac1 and modulates their activity. Activation levels of CB1 receptor directly impacted on actin polymerization and stability via WAVE1 in growth cones of developing neurons, leading to their collapse, as well as in synaptic spines of mature neurons, leading to their retraction. In adult mice, CB1 receptor agonists attenuated activity-dependent remodeling of dendritic spines in spinal cord neurons in vivo and suppressed inflammatory pain by regulating the WAVE1 complex. This study reports novel signaling mechanisms for cannabinoidergic modulation of the nervous system and demonstrates a previously unreported role for the WAVE1 complex in therapeutic applications of cannabinoids.

## Abstract 2

A proteomic study reveals the actin nucleation complex WAVE1 as a hitherto unknown binding partner of cannabinoid receptor 1 and explores the functional role of this interaction in regulating pain-related structural plasticity.

## Author Summary

One of the most interesting features of the endocannabinoid system (a group of neuromodulatory lipids and their receptors, which promotes homeostasis in a variety of physiological processes) is its ability to counteract nociception or pain. This function is largely mediated by the receptor component of the endocannabinoid system. One of the most-studied types of cannabinoid receptors, the cannabinoid receptor 1 (CB1R), exerts its antinociceptive function at all levels of the central nervous system, from the periphery up to the brain. Despite numerous studies on the role of CB1R and its antinociceptive effect, our knowledge of the molecular mechanisms underlying this particular feature is still lacking. In this study, we identify the WAVE1-complex—known to be involved in actin nucleation—as novel interacting partners of CB1R. We observe a functional relationship between the WAVE1-complex and CB1R in the regulation of actin filaments in developing as well as mature cultured neurons. Furthermore, we show that inflammation-induced structural plasticity in spinal neurons that contributes to hyperalgesia is regulated by CB1R in a WAVE1-dependent fashion. These findings expand our understanding of CB1R signaling and of the physiological as well as pathological context of pain.

## Introduction

Psychoactive cannabinoids derived from marijuana as well as diverse endocannabinoids, such as anandamide and 2-arachidonylglycerol (2-AG) bind to and activate signaling via cannabinoid type 1 and type 2 (CB1 and CB2) receptors, which belong to the G-protein coupled receptor (GPCR) family. Gene deletion studies have uncovered the tremendous significance of the CB1 receptor in mediating several key biological functions of (endo)cannabinoids in the adult central nervous system (CNS), including learning and memory, pain and analgesia, neuronal excitability and seizures, fear acquisition and extinction, and appetite control, amongst several others [1–3].

CB1 receptor is abundantly expressed in inhibitory interneurons at presynaptic terminals, where it is activated by retrogradely acting endocannabinoids synthetized at the postsynaptic site [4,5]. CB1 receptor is also found to be expressed at more moderate levels in excitatory neurons (principle cells) in the CNS. Cell type-specific CB1 receptor knockout mice have particularly revealed key roles for CB1 receptor expressed in excitatory neurons in appetite, neuroprotection, epilepsy, fear, anxiety, pain, and analgesia, amongst others [6–10]. Recent studies have shown that far from enfolding their functions in the adult nervous system alone, endocannabinoids are important regulators of neuronal development; particularly, the CB1 receptor has been implicated in collapse and navigation of axonal growth cones in inhibitory as well as excitatory neurons [11–13].

Interestingly, the vast knowledge on the biological functions of endocannabinoids as well as exogenously-applied cannabinoids at the cellular and systemic levels [1,14] is contrasted sharply by the relative scarcity of studies addressing molecular components of cannabinoid receptor assembly and signaling partners. CB1 receptor primarily couples to G_i_ but can also couple with G_q/11_ or G_12/13_ in a context-dependent manner, which have primarily represented the focus of previous studies [15–17]. To date, however, very little is known about which other proteins assemble with CB1 receptor in a multiprotein signaling complex to unfold its biological actions. The few interactions that were tested on a candidate protein basis pertain largely to trafficking and chaperone proteins modulating the endocytosis and intracellular trafficking of CB1 [18–22]. There are, to date, no reports on unbiased proteomic screens for CB1 receptor in neural tissue. Indeed, proteomic screens on GPCRs are notoriously challenging because of technical difficulties in solubilizing GPCR protein complexes.

This study now reports novel members of the CB1 receptor complex found via a proteomic screen in mouse brain in vivo. We particularly report on the functional significance of CB1 receptor interactions with the Wiskott-Aldrich syndrome protein-family verprolin-homologous protein 1 (WAVE1)/SCAR1 complex. WAVE proteins act as nucleation promoting factor linking upstream signals to the activation of actin related protein 2/3 (ARP2/3), which in turn activates actin nucleation [23,24]. Using a diverse set of models and in vitro as well as in vivo analyses, we demonstrate that CB1-WAVE1 interactions play a key role in dynamically regulating the actin cytoskeleton in developing and adult neurons, which contribute to prominent functions of endocannabinoids in the brain and spinal cord. Importantly, we demonstrate that cannabinoids structurally remodel synaptic spines by regulating the activity levels of WAVE1 and report a novel function for WAVE1 in mediating inflammatory pain via structural and functional plasticity of spinal neurons.

## Results

### Proteomic Screen on Cortical Neurons In Vivo Identifies Interactions of CB1 Receptor with the WAVE1 Complex

**Fig 1:**
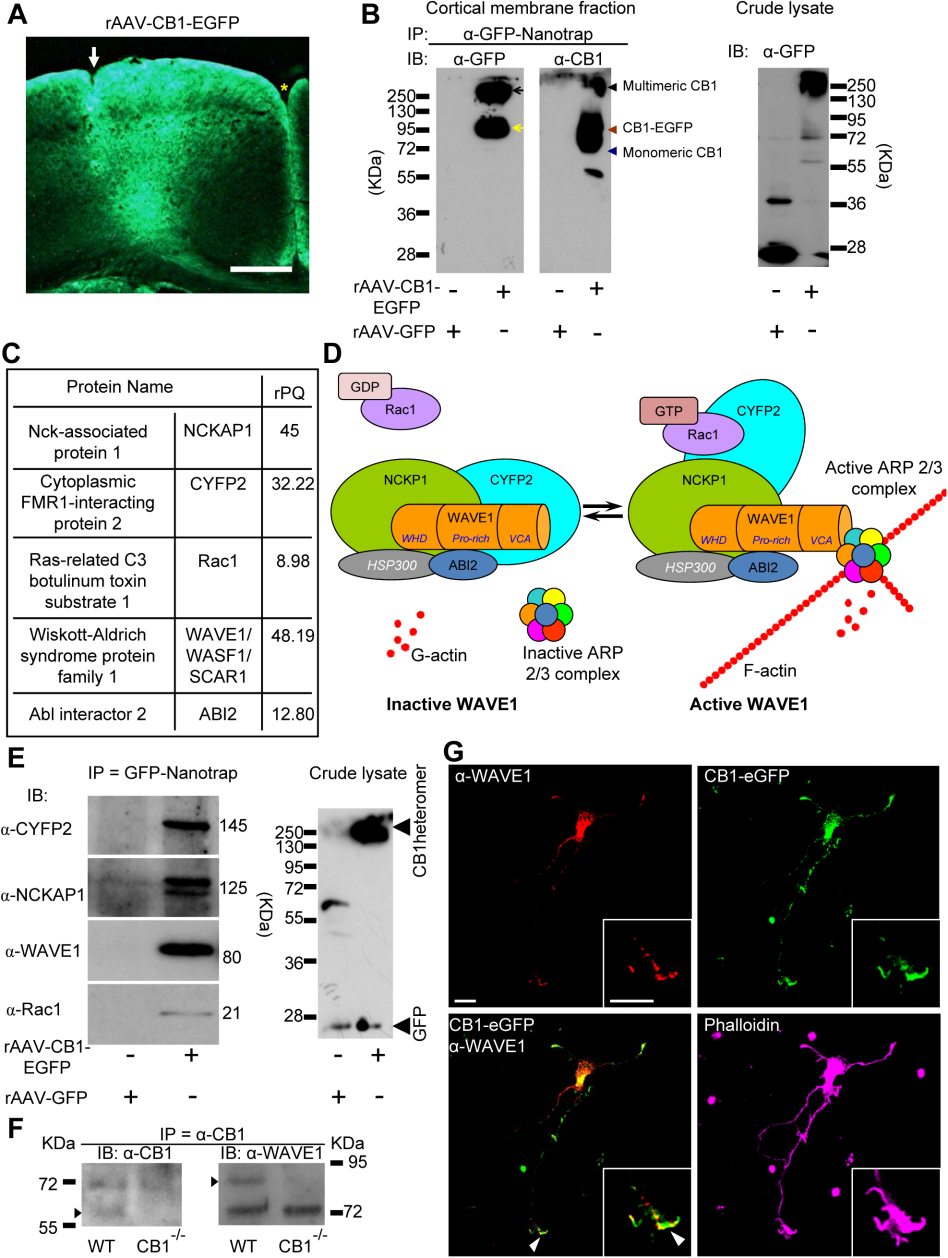
CB1 physically interacts with members of the WAVE1 signaling complex in mouse brain and colocalizes with WAVE1 in neurons. (A) Expression of EGFP-tagged CB1 (CB1-EGFP) in mouse brain 4 wk after cortical injection of rAAVs. Injection site is marked with a white arrow, and medial longitudinal fissure with a yellow asterisk. Scale bar represents 0.5 mm. (B) Validation of solubilization and pulldown of CB1 by immunoprecipitation using GFP-nanotrap on membrane fractions derived from cortex of mice expressing either CB1-EGFP or GFP alone (as control). Immunoblotting (IB) experiments show that anti-GFP antibody pulls down endogenous CB1 and CB1-EGFP from CB1-EGFP-expressing mice, but not from GFP control mice. The successful pulldown shows the high molecular weight form of CB1 (black arrows), the CB1-EGFP monomer (yellow arrow) and endogenous CB1 (blue arrow head). (C) Summary of MS analysis of GFP nanotrap-immunoprecipitates from the cortex of CB1-EGFP-expressing mice or GFP-expressing control mice (*n* = 5). rPQ is relative peptide query score. Values for rPQ > 4 indicates specific purification in comparison over negative control. (D) Schematic representation of activation of the WAVE1 complex by GTP-bound (activated) Rac1. (E) Immunoblotting on GFP-nanotrap-immunoprecipitates showing that cytoplasmic FMR1 interacting protein 2 (CYFIP2), NCK-associated protein 1 (NCKAP1), WAVE1 and Rac1 are coimmunoprecipitated with GB1-EGFP, but not with GFP, from the mouse cortex. (F) Immunoblotting on α-CB1-immunoprecipitates showing that WAVE1 is coimmunoprecipitated with CB1 from cortical lysates derived from wild-type mice, but not in lysates from CB1-deficient mice (CB1^-/-^). (G) Colocalization of WAVE1 and EGFP-tagged CB1 in growth cones (magnified in inset) of developing cortical neurons with pyramidal morphology. The actin cytoskeleton is counterstained with Phalloidin (growth cone magnified in inset). Scale bars represent 10 μm.

A proteomic strategy combining immunoprecipitation and mass spectrometry (MS) was used to identify new potential interacting partners of CB1 receptor. Owing to limited success of currently available antibodies against CB1 receptor in immunoprecipitation experiments, we generated recombinant adeno-associated virions (rAAV) expressing enhanced green fluorescent protein (EGFP)-tagged CB1 (rAAV-EGFP-CB1), in which the N-terminus is fused to EGFP, which we have previously shown to bind and respond to cannabinoids in an identical manner as wild-type CB1 receptor [22]. rAAV-EGFP-CB1 virions were stereotactically injected into the frontal and parietal cortex of adult mice (Fig 1A) and yielded a strong expression of EGFP-CB1 in cortical neurons, which was mostly localized to the neurophil, consistent with the targeting of CB1 receptor to axonal and dendritic segments [25]. We then proceeded to optimize membrane protein-enriched preparations and test a large number of detergents in varying concentrations and combinations to obtain a good solubilization of EGFP-CB1 receptor from cortical membranes, which is important for successful and specific pull-down of interacting protein assemblies without destroying protein–protein interactions in a functional complex. Western blot analysis on the immunoprecipitate of the solubilized fractions using a GFP-nanotrap antibody [26] revealed a single band around 250 KDa in CB1-EGFP-transfected HEK293 cell lysates following solubilization with Na-cholat (S2A Fig), which represents the multimeric state of the CB1 receptor [27]. These optimized conditions, when applied to rAAV-EGFP-tagged CB1-transduced brain tissue, yielded high molecular weight forms of CB1 receptor when blotted against GFP and CB1 receptor (Fig 1B, left blot, black arrow). Additionally, a band at approximately 95 KDa was also observed in pulldowns from brain tissue (Fig 1B, left blot, yellow arrow), corresponding to the size of monomeric CB1-EGFP (CB1: 64 KDa and EGFP: 28 KDa). Immunoblotting with an anti-CB1 receptor antibody additionally yielded a band just below 70 KDa, indicating immunoprecipitation of the endogenous CB1 receptor monomer (Fig 1B, middle blot, blue arrowhead). We observed GFP protein in the lysates from rAAV-GFP transduced (control) brain tissue, but not in the immunoprecipitated material (Fig 1B right blot).

GFP-nanotrap-immunoprecipitated materials from solubilized mouse cortex preparations were then analyzed by high-resolution nanoflow liquid chromatography tandem MS (nano-LC MS/MS) in 5 independent immunoprecipitation experiments (a comprehensive list of all proteins identified in the MS analyses as potential CB1 interactors is given in S1 Table). Apart from CB1-EGFP and endogenous CB1 receptor, several known interacting partners of CB1 receptor, such as adenylate cyclase [28], cannabinoid receptor-interacting protein 1 [20], clathrin coat assembly proteins [29], and guanine nucleotide-binding protein G_q_ [17] were coimmunoprecipitated by GFP-nanotrap with high relative peptide query (rPQ) in rAAV-CB1-EGFP-mice, but not in rAAV-GFP-expressing control mice (S1B Fig; loading control S1A Fig; S1 Table).

Importantly, several members of the Wiskott-Aldrich syndrome protein family verprolin homologous protein 1 (WAVE1) complex and its upstream signaling components were consistently coimmunoprecipitated with CB1 receptor with a high specificity in rAAV-CB1-EGFP-expressing mice, but not in rAAV-GFP-expressing mice. The WAVE1 complex consists of WAVE1 (also known as SCAR1), Abelson-interacting protein 1/2 (ABI1/2), NCK-associated protein 1 (NCKAP1, also known as NAP1), cytoplasmic FMR1-interacting protein 2 (CYFIP2, also known as PIR121 or SRA1) and HSPC300 [30]. Under basal conditions, proteins of the WAVE1 complex are positioned to block the VCA domain of WAVE1, thereby inhibiting WAVE1 activity towards ARP2/3 (see scheme in Fig 1D, left image). This autoinhibition is then released upon binding with the active form of the RhoGTPases, Rac1, leading to the activation of ARP2/3 by WAVE1 and thereby to actin nucleation [31] (Fig 1D right image). Our analyses revealed that four constituents of this protein complex, namely WAVE1, ABI1/2, NCKAP1, and CYFIP2, as well as the upstream activator, Rac1, were coimmunoprecipitated with solubilized CB1 (Fig 1C).

We confirmed results from MS experiments by blotting immunoprecipitates with antibodies recognizing diverse components of the WAVE1 complex. Specific bands corresponding to WAVE1, CYFIP2, NCKAP1, and Rac1 were observed in samples coimmunoprecipitated by the GFP nanotrap in rAAV-EGFP-tagged CB1-expressing mice but not in rAAV-GFP-expressing mice (Fig 1E). Using HEK293 cells heterologously transfected with CB1-EGFP or GFP alone for validation of the above results, we observed that the anti-EGFP antibody pulled down endogenous WAVE1 from cells expressing CB1-EGFP, but not from cells expressing GFP alone (S2A Fig). Finally, we additionally validated the interaction in native brain tissue by immunoprecipitating endogenously expressed CB1 from wild-type mouse cortex. We observed that WAVE1 protein was coimmunoprecipitated with endogenous CB1 when lysates of wild-type mouse cortex were employed, but not in lysates derived from mice lacking CB1 in a global manner (CB1^-/-^ mice) [32], thereby establishing in vivo relevance as well as specificity of the interaction (Fig 1E).

In primary cortical neurons cultured from E15 mouse embryos infected with rAAV-CB1-EGFP virions and analyzed at 3 d in vitro, a stage at which they are still developing, marked overlap was observed between CB1-EGFP and anti-WAVE1 immunoreactivity in the somata and growth cones of processes (Fig 1F, note the common domain occupied by CB1-EGFP and WAVE1 at the leading edge within the broad growth cone structure labelled via Phalloidin staining of actin in the inset in Fig 1F). Importantly, in nontransfected primary embryonic cortical neurons, WAVE1 also colocalized with native anti-CB1 immunoreactivity, the latter being in axonal growth cones, in which CB1 was markedly found in the developing axons (S2B Fig) as reported elsewhere [11].

**Fig 2:**
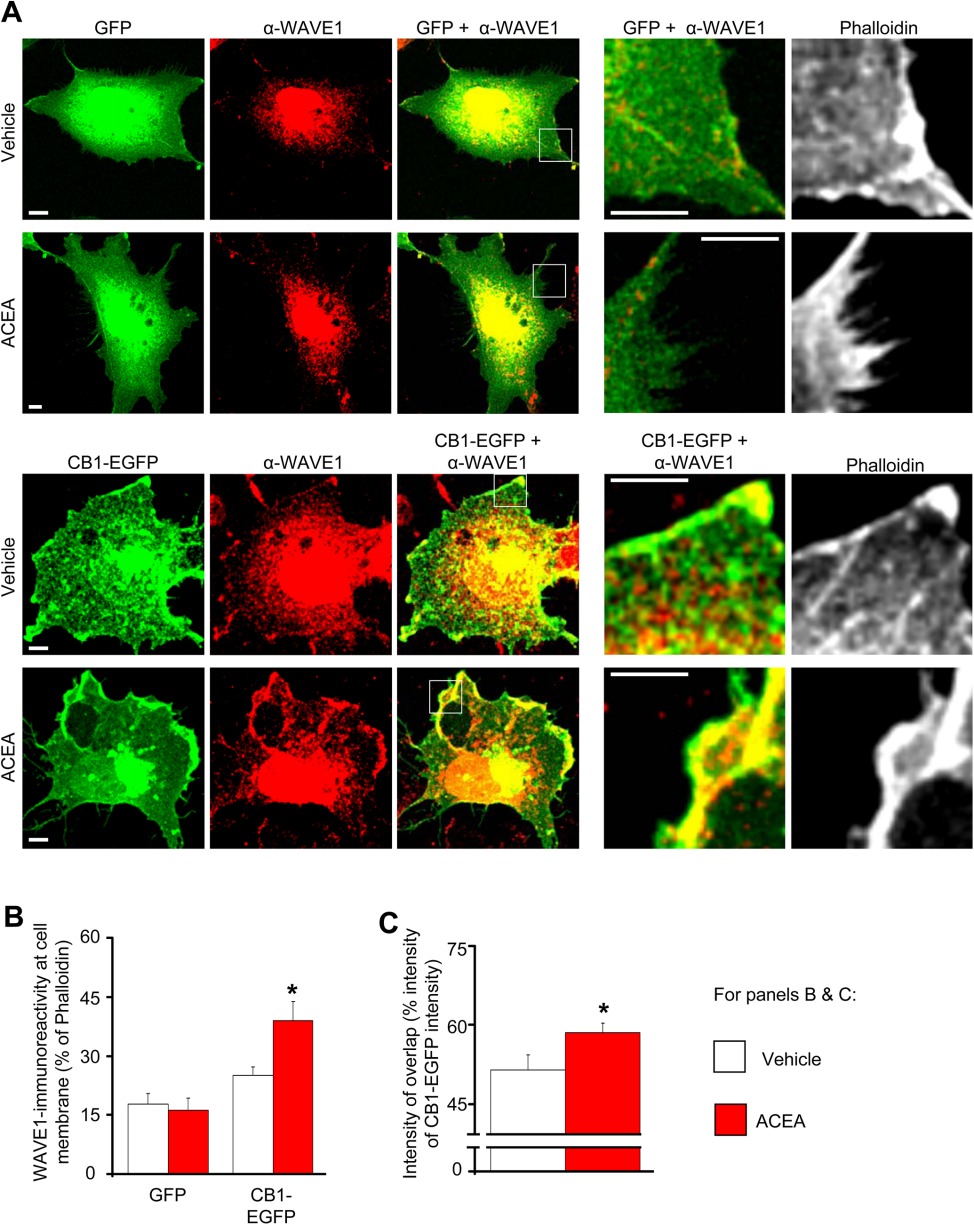
Increased localization of colocalization of WAVE1 and CB1-EGFP at the cell membrane in heterologously-transfected COS7 cells upon ACEA treatment. (A) Distribution of heterologously-transfected WAVE1 in cells cotransfected with either GFP (control, upper panels) or CB1-EGFP (lower panels) following treatment with vehicle (DMSO 1:30,000) or CB1 agonist (ACEA 100 nM) for 45 min. Scale bar represents 5 μm. (B) Quantitative summary of intensity of WAVE1-immunoreactivity relative to Phalloidin-stained actin at the plasma membrane in GFP- or CB1-EGFP-coexpressing COS7 cells treated with vehicle (white bars) or ACEA (red bars). (C) Quantitative summary of CB1-EGFP-WAVE1 colocalization in vehicle- or ACEA-treated cells calculated as a fraction of the total intensity of CB1-EGFP fluorescence. Values in panels B and C represent the mean ± SEM and are derived from analyses on at least 15 COS7 cells each over several independent culture experiments. *p < 0.05 one way ANOVA followed by posthoc Tukey’s test.

To further test colocalization of WAVE1 and CB1, we exploited the large size and elaborate morphology of COS (**C**V-1 in **O**rigin with **S**V40 genes) cells. In COS7 cells cotransfected with WAVE1 and GFP (control), heterologously-transfected WAVE1 showed a highly specific distribution, which was predominantly nuclear and perinuclear, with a much lesser degree of localization at the cell membrane than the cell interior (Fig 2A, upper panels). In cells cotransfected with CB1-EGFP and WAVE1, CB1-EGFP colocalized with WAVE1 in the cell membrane as well as in the cytoplasm, the latter being the predominant locus of overlap (Fig 2A, lower panels). However, in CB1-EGFP-transfected COS cells treated for 45 min with the CB1 agonist, arachidonyl-2’-chloroethylamide (ACEA), there was a marked increase in the proportion of WAVE1 localized at the cell membrane (Fig 2A lower panels; quantitative analyses from several experiments are shown in Fig 2B), and there was a significant increase in colocalization of WAVE1 and CB1 (inset in lower panels of Fig 2A; quantitative summary in Fig 2C). In cells cotransfected with WAVE1 and GFP (control), no significant change was observed in the localization of WAVE1 at the cell membrane in ACEA-treated and vehicle-treated groups (Fig 2A upper panels; Fig 2B). This suggests that activated CB1 and WAVE1 colocalize in the cell membrane and that activation of CB1 redistributes WAVE1 towards the cell membrane.

### Cannabinoids Functionally Modulate the Activity of Rac1 and the WAVE1 Complex

**Fig 3:**
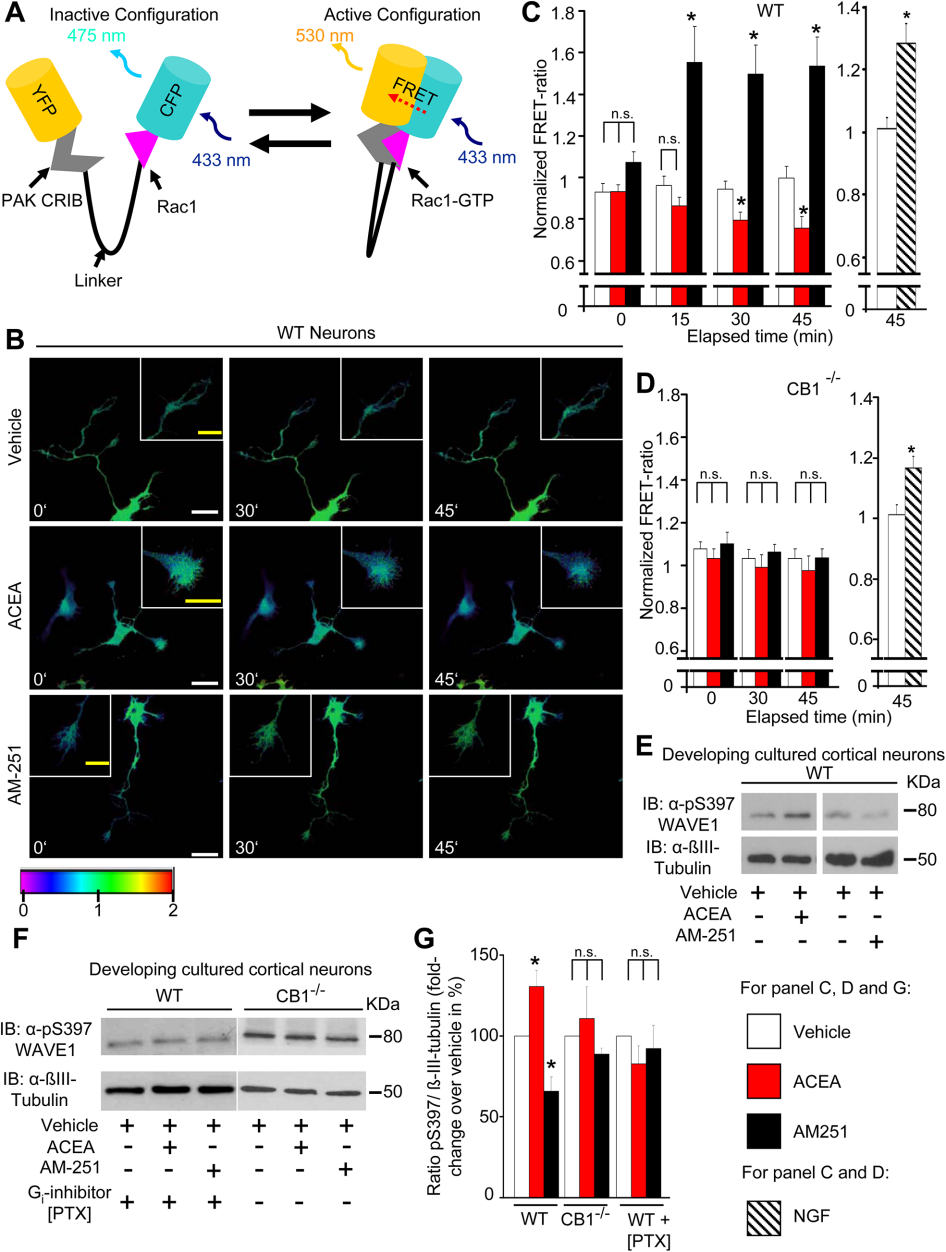
Cannabinoids directly modulate Rac1 activity and WAVE1 phosphorylation by Rac1 Activation and WAVE1 Phosphorylation via CB1. (A) Schematic representation of the Raichu-Rac1 FRET-biosensor employed to measure Rac1 activity. (B) Real-time images representing changes in Rac1 activity in developing mouse cortical neurons following treatment with a CB1 agonist (ACEA; 100 nM) and an inverse agonist at CB1 (AM251; 600 nM). The FRET signal intensity is represented as a pseudocoloured heat map, and insets show magnified view of growth cones. Scale bars (white) represent 20 μm, and scale bars in the inset (yellow) represent 5 μm. (C) Quantitative summary of normalized FRET ratios over the growth cone area at various time points after addition of ACEA, AM251, NGF (100 ng/ml), or vehicle normalized to the average FRET ratio value over the same area prior to addition of pharmacological agents in developing neurons derived from wild-type mice. (D) Preserved effect of NGF and loss of effects of ACEA as well as AM251 on Rac1 activity in developing cortical neurons derived from CB1^-/-^ mice. Values in panels C and D represent the mean ± SEM and are derived from analyses on 10–16 neurons per group over at least three independent culture experiments. (E, F) Immunoblot analyses showing changes in phosphorylation state of Serine 397 (pSer397) in WAVE1 upon treatment with ACEA (100 nM) or AM251 (600 nM) as compared to vehicle treatment in cortical neurons derived from wild-type mice without pretreatment (E), with overnight pertussis toxin (PTX) (100 ng/ml) pretreatment or from CB1^-/-^ mice (F). (G) Quantitative summary of cannabinoid-induced modulation of pSer397 WAVE1 levels normalized to βIII-tubulin in the above groups (*n* = 5–6 independent culture experiments). All graphs represent mean values ± SEM *p < 0.05, two-way ANOVA for repeated measures (C, D) or one-way (G) ANOVA followed by posthoc Tukey’s test. N.s. stands for not significant.

It is well-recognized that Rho-family GTPases, to which Rac1 belongs, are subject to tight spatiotemporal regulation, and that multiple subcellular pools of any given Rho GTPase can operate simultaneously yet independently of each other in both time and space [33]. Therefore, instead of globally pulling down Rac1 from cell lysates, we spatiotemporally imaged Rac1 activity in growth cones of cortical neurons nucleofected with a biosensor (Raichu-Rac1 [34]), a construct consisting of YFP, a ligand domain (PAK CRIB), a flexible linker peptide, a sensor domain (Rac1) and CFP (Fig 3A, typical examples of fluorescence resonance energy transfer [FRET] expression in neurons shown in S3A Fig). Binding of Rac1 to GTP leads to FRET of excitation energy from CFP to YFP, i.e., the active state of Rac1 corresponds to an increase in YFP to CFP fluorescence ratio (FRET ratio; Fig 3A, right image). Although bleaching leads to a small decrease in total fluorescence intensity over time, the FRET ratio stays constant [34], as observed upon treatment of neurons with vehicle over 45 min (typical examples in Fig 3B and quantitative summary from ten neurons from five independent cultures is shown in Fig 3C). Nerve growth factor (NGF), which activates Rac1 in growth cones [35], served as a positive control, and was observed to evoke a significant increase in FRET ratio over 1 h treatment, as compared to vehicle (DMSO, end-dilution 1:30,000 in neurobasal medium; Fig 3C and S3B Fig). Using this model system, we observed that the CB1 receptor agonist (ACEA) or inverse agonist (AM251) bidirectionally altered the activity of Rac1 in growth cones of cortical neurons (typical examples in Fig 3B and quantitative summary from 10–16 neurons from five independent cultures at 0, 15, 30, and 45 min post-treatment is shown in Fig 3C; detailed time course over 45 min is shown in S3C Fig). Upon application of the agonist ACEA, growth cones showed a gradual decrease in FRET signals, reaching significant at 30 min and further progressively decreasing in magnitude until 45 min (Fig 3C and S3C Fig). In contrast, the CB1 receptor inverse agonist, AM251, induced a rapid and marked increase in the FRET ratio within a few minutes of treatment (see time course in S3C Fig), which stayed constant over 45 min of analysis (Fig 3C). Importantly, neither ACEA nor AM251 induced any significant changes in FRET ratio in growth cones of cortical neurons cultured from CB1^-/-^ mice (Fig 3D), although NGF-induced increase in FRET ratio was preserved in CB1^-/-^ neurons (Fig 3D and S3D Fig), thereby validating that the changes in Rac1 activity observed in wild-type neurons are specific and receptor-dependent. No changes were observed in FRET ratios over somata upon treatment with ACEA, AM-251, or NGF as compared to vehicle (S3D Fig), consistent with lack of morphological changes in the somata in response to these agents.

Given that Rac1 activity is critically linked to disinhibition of WAVE1 signaling onto the Arp2/3 complex, these results predicted that agonism and inverse agonism at CB1 receptor bidirectionally modulate WAVE1 activity. We then studied the extent of serine phosphorylation in WAVE1 as a surrogate parameter for the activation status of WAVE1. WAVE1 can be phosphorylated on three serine sites, namely S310, S397, and S441, corresponding to an inhibited state and the dephosphorylated state has been linked to WAVE1 activation [36]. In developing primary cortical neurons, WAVE1 showed basal levels of phosphorylation on Ser397 (pS397-WAVE1) and treatment with a CB1 receptor-specific agonist ACEA (100 nM) significantly enhanced the proportion of pS397-WAVE1 over basal state as well as in comparison to vehicle. Representative example of Western blot analysis of pS397-WAVE1 and β-tubulin (loading control) are shown in Fig 3E, and the corresponding western blot analysis of total WAVE1 expression is shown in S3E Fig. Quantitative summary from at least four independent experiments of pS397-WAVE1 as a function of β-tubulin levels or total WAVE1 levels is shown in Fig 3G and S3E Fig, respectively. Conversely, treatment with CB1 receptor inverse agonist AM251 (600 nM) significantly decreased the proportion of pS397-WAVE1 over basal as compared to vehicle treatment (Fig 3G and S3E Fig). Neither ACEA nor AM251 affected pS397-WAVE1 levels in cortical neurons cultured from CB1^-/-^ mice (Fig 3F and Fig 3G) or in wild-type neurons pretreated with pertussis toxin (PTX) (100 ng/ml), a specific inhibitor of G_i_ signaling (Fig 3F and Fig 3G). These results thus show that a bidirectional change in CB1 receptor activity leads to a corresponding, Gi-dependent bidirectional alteration in the serine phosphorylation status of WAVE1.

### Cannabinoids Dynamically Regulate the Actin Cytoskeleton in Growth Cones of Cortical Neurons via the WAVE1 Complex

**Fig 4:**
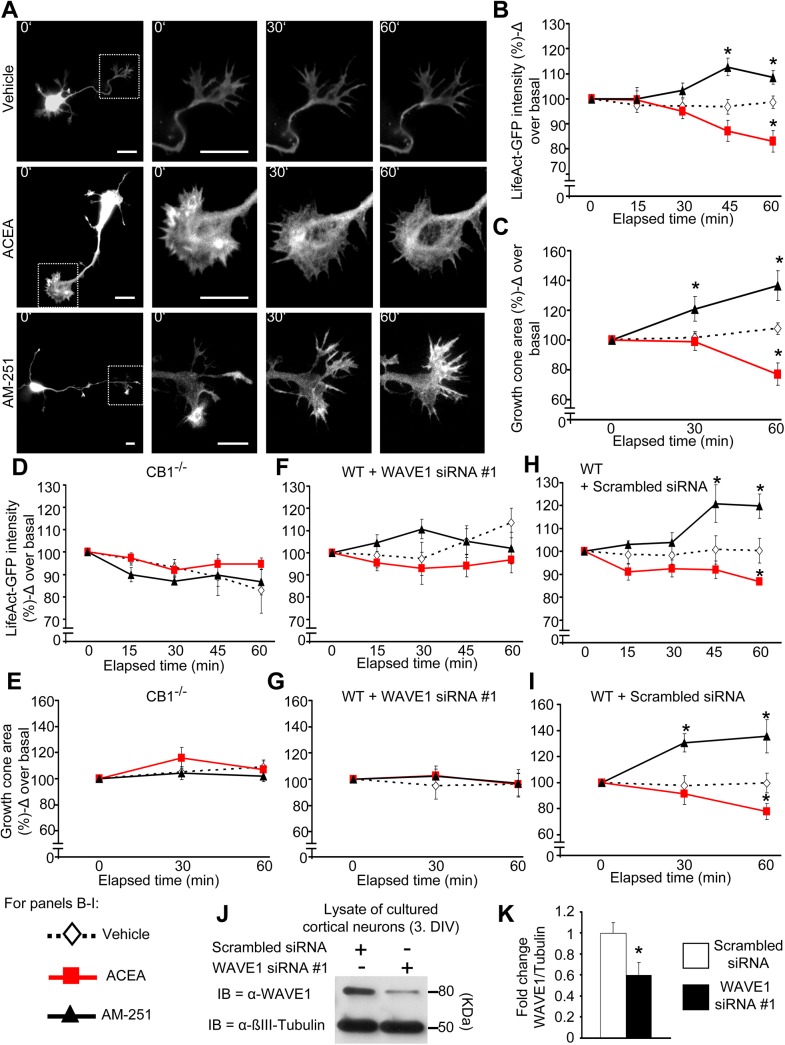
Visualization of cannabinoidergic modulation of actin dynamics in neuronal growth cones mediated by CB1 and WAVE1. (A) Real time images of wild-type developing cortical neurons transfected to express LifeAct-GFP, a biosensor for levels of F-actin prior to and at different time points following treatment with ACEA (100 nM), AM251 (600 nM), or vehicle. Scale bar represents 10 μm. (B, C) Analysis on the F-actin dynamics based on cannabinoid-induced changes in LifeAct-GFP intensity (B) and area (C) over time in growth cones of cultured cortical neurons derived from wild-type mice (*n* = 15–17 neurons per group from 5 independent culture experiments). (D, E) However, no changes in LifeAct intensity (D) nor area of the growth cones (E) were observed with cultured cortical neurons derived from CB1^-/-^ mice (*n* = at least 4 neurons per group from 2 independent culture experiments) after treatment. (F, G) Similarly, cultured cortical neurons with down-regulated WAVE1 showed no changes in LifeAct intensity (F) or area of growth cones (G) after cannabinoid treatment (*n* = at least 4 neurons per group). (H, I) Moreover, the abrogation of the effects after cannabinoid treatment can be contributed to the down-regulation state of WAVE1, since cultured cortical neurons nucleofected with scrambled siRNA, in turn show changes in LifeAct intensity (H) and area (I) of the growth cone following cannabinoid treatment (*n* = at least 4 neurons per group). (J, K) Immunoblot representation of WAVE1 down-regulation upon siRNA delivery in developing cortical neurons (J) and the corresponding quantification (K) (*n* = 6). All graphs represent mean values ± SEM *p < 0.05, two-way ANOVA (B-I) or one-way ANOVA (K) for random measures followed by posthoc Tukey’s test.

We employed LifeAct-GFP, which constitutes the first 17 amino acids of Abp140 protein tagged with GFP and preferentially binds to filamentous actin (F-actin) rather than actin monomers (G-actin) and thereby permits a direct visualization of F-actin [37]. In LifeAct-GFP-transfected developing cortical neurons (examples shown in Fig 4A), we observed that intensity of labeled F-actin decreased gradually upon exposure to ACEA over basal (preapplication) values, reaching significance over basal values at 60 min following ACEA application (Fig 4B). In contrast, vehicle-treated neurons demonstrated comparable intensity over 60 min, indicating that ACEA-induced decrease in LifeAct-GFP intensity stems from F-actin destabilization rather than from bleaching. Moreover, the area covered by F-actin shrunk significantly upon ACEA treatment, in contrast to vehicle (Fig 4C). Conversely, AM251 treatment led to an expansion of the area covered by F-actin within 30 min postapplication and enhanced the intensity of F-actin (Fig 4B and Fig 4C), thus indicating enhanced stability of F-actin and actin polymerization.

In contrast to wild-type neurons (above), ACEA and AM251 did not affect actin dynamics in developing cortical neurons cultured from CB1^-/-^ mice (Fig 4D and Fig 4E). Furthermore, we observed that both modes of actin modulation by cannabinoids are lost upon down-regulation of WAVE1 expression. In cultured cortical neurons nucleofected with WAVE1-specific siRNA (Fig 4J and Fig 4K; 59.6 ± 12.5% knockdown as compared to scrambled siRNA), we observed that neither ACEA nor AM251 affected actin dynamics in contrast to neurons transfected with scrambled siRNA (Fig 4F to Fig 4I). Because siRNAs can have significant off-target effects, we implemented an independent siRNA#2; targeting WAVE1 at an independent locus as compared to the siRNA#1 employed above would be important for ruling out off-target effects of the formerly-used WAVE1 siRNA (Materials and Methods). We verified the degree of WAVE1 knockdown with siRNA via western blot analysis (example in S4A Fig and quantitative summary in S4B Fig) and, in Life-Act-GFP assays, observed exactly the same phenotypic effects as siRNA#1 (S4C Fig), establishing that these are indeed caused by WAVE1 down-regulation. Thus, WAVE1 links CB1 receptor with modulation of actin dynamics in developing neurons.

### Bidirectional Modulation of Growth Cone Morphology by Cannabinoid Agonists and Inverse Agonists Acting via CB1 and WAVE1

**Fig 5:**
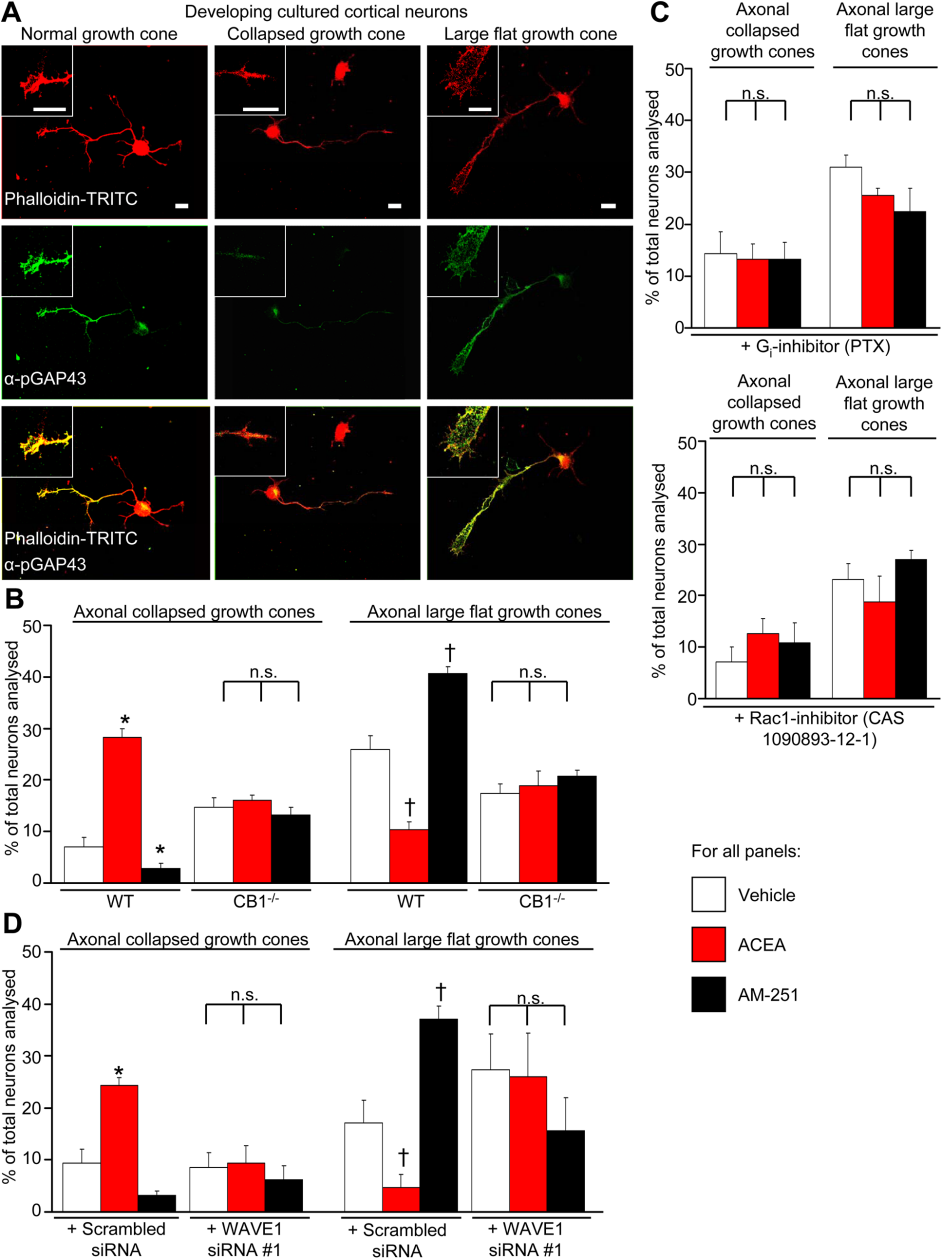
Cannabinoids regulate growth cone morphology via CB1-Gi-Rac1-WAVE1 signaling. (A) Characterization of changes in growth cone morphology in developing cultured cortical neurons immunostained with anti-pGAP43 to recognize normal or enlarging growth cones and Phalloidin to label actin. Insets show normal, enlarged, or collapsed morphology in developing cortical neurons; scale bar represents 10 μm in all images and insets. (B) Bidirectional modulation of the proportion of growth cones with collapsed or enlarged morphology upon treatment with ACEA (100 nM), AM251 (600 nM) or vehicle (DMSO 1:30,000) for 1 h in neurons derived from wild-type mice (*n* = 10 independent culture experiments), but not in neurons from CB1^-/-^ mice (*n* = 4). (C–F) Lack of modulation of cannabinoidergic modulation of growth cone morphology upon treatment in the presence of PTX (100 ng/ml), Rac1 inhibitor, CAS 1090893-12-1 (50 μM) or in neurons with siRNA-mediated downregulation of WAVE1; control siRNA-transfected neurons showed significant cannabinoidergic modulation (*n* = 4). All graphs represent mean values ± SEM. *p < 0.05, one-way ANOVA followed by posthoc Tukey’s test. N.s. stands for not significant.

Activation of CB1 receptor has been reported to induce growth cone collapse in developing GABAergic neurons [12] as well as in excitatory neurons of the cortex [11]. We treated developing cultured cortical neurons with cannabinoids or vehicle for 1 hr and identified axonal growth cones via staining for Tau 1 (axonal marker) and Phalloidin (labels actin), as well as phosphorylated growth-associated protein 43 (pGAP43), which is expressed in stable and expanding growth cones, but not collapsing or retracting growth cone [38]. As described previously, axonal growth cones could be categorized into three different morphologies (illustrated by examples shown in insets in Fig 5A): 1) normal or stable growth cone, characterized by an expanding cone larger than its proximal area and existence of pGAP43 staining (seen in about 66.9 ± 3.4% of vehicle-treated neurons), 2) collapsed growth cone, characterized by a thin-shaped cone and lack of pGAP43 staining (seen in about 7.1 ± 1.7% of vehicle-treated neurons), and 3) a large flat growth cone, characterized by the enlarged filopodia-rich rounded shape of the cone and strong pGAP43 staining (seen in about 26 ± 2.7% of vehicle-treated neurons) (Fig 5B). We observed that a significantly higher number of ACEA-treated neurons showed collapsed morphology as compared to vehicle-treated neurons (Fig 5B). In contrast, inverse agonism at CB1 receptor led to a further significant decrease in the proportion of neurons showing collapsed growth cones (Fig 5B). Conversely, the incidence of large flat growth cones was significantly higher in AM251-treated neurons and significantly lower in ACEA-treated neurons as compared to vehicle (Fig 5B).

Modulation of growth cone morphology by ACEA and AM251 was not observed in cortical neurons derived from CB1^-/-^ mice (Fig 5B) and was also inhibited in wild-type neurons pretreated with a G_i_ inhibitor, PTX (100 ng/ml) or a Rac1 inhibitor, CAS 1090893-12-1 (50 μM) as compared to the respective vehicles (Fig 5C). Importantly, neurons with markedly reduced expression of WAVE1 via siRNA-mediated knockdown did not show significant effects on growth cone morphology in contrast to neurons expressing scrambled (control) siRNA (Fig 5D).

Thus, taken together, these results reveal causal relationships between CB1 receptor signaling, the WAVE1 complex, actin rearrangement and structural modulation in developing neurons.

### Expression of Endogenous CB1 and CB1-EGFP in Axons as well as Dendritic Compartments, Including Dendritic Spines, in Mature Primary Cortical Neurons and Spinal Cord Neurons

To further study the impact of CB1-WAVE1 interactions on neuronal morphology, we employed cortical neurons matured over 4 wk in vitro. Although CB1 receptors are believed to be mainly expressed presynaptically, in axonal varicosities, several studies have previously reported that CB1 can be additionally localized in dendrites of neurons [39–41].

**Fig 6:**
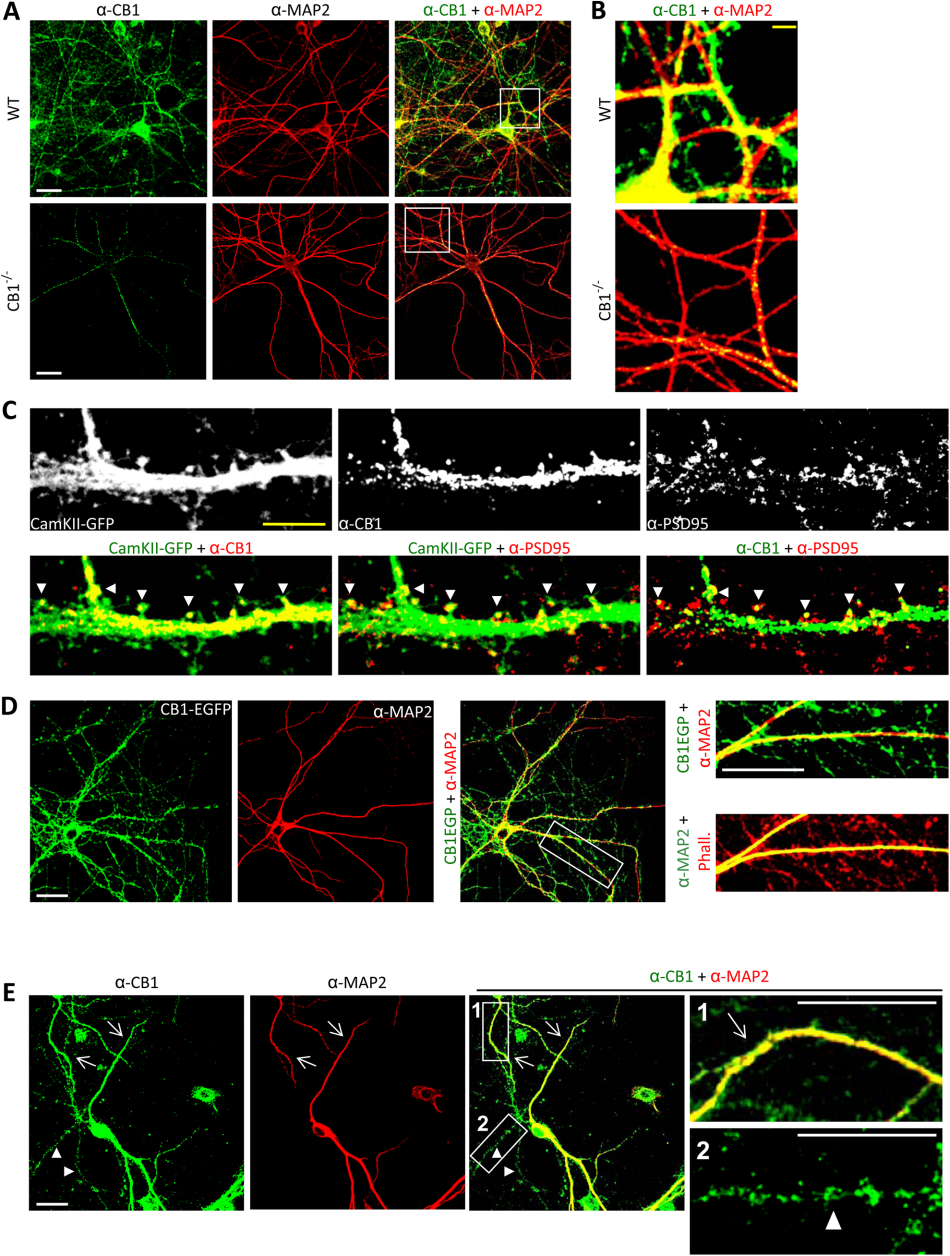
CB1 receptor is expressed in axonal as well as dendritic compartments of primary neurons matured over 4 wk in vitro. (A) Coimmunostaining against CB1 receptor and dendritic marker MAP2 on 28 days in vitro (DIV) cortical neurons derived from wild-type mouse embryos (upper panels) shows the distribution of CB1 receptor in the axonal compartments (MAP2-negative) as well as dendritic compartments (MAP2-positive). The rabbit anti-CB1 antibody used throughout this experiment did not yield substantial staining in cortical neurons cultured from CB1-deficient mouse embryos at 28 DIV (lower panels). (B) Magnified images represent white boxed areas from main images. (C) CB1 receptor colocalizes with CamKII-GFP and the synaptic protein PSD95 in dendritic compartments, including several spine heads (white arrow heads), indicating postsynaptic localization. (D) Heterologously-expressed CB1-EGFP in mature cultured cortical neurons shows axonal and dentritic localization upon costaining with MAP2. Phalloidin is used to counterstain the entire actin cytoskeleton. Phall. stands for Phalloidin. (E) Dendritic (arrows) ad axonal (arrowhead) localization of endogenous CB1 in cortical neurons cultured from wild-type mice and matured at 28 DIV. White scale bars represent 20 μm and yellow scale bars represent 5 μm.

To ascertain the expression pattern of CB1 in mature primary neurons, we studied the distribution of endogenously expressed CB1 (Fig 6A) using a previously well-characterized antibody ([10]; lack of staining in CB1^-/-^ mice is shown as a negative control in Fig 6A, lower panel). In addition to the widespread distribution of CB1 in axonal compartments (negative for the dendritic marker protein, MAP2), these neurons showed a marked distribution of CB1 in MAP2-positive dendrites. Both dendrites and axons showed hot spots of CB1 expression, reminiscent of varicosities and dendritic spines (see overlay in Fig 6A, right column and Fig 6B). Indeed, when we infected mature cortical neurons with rAAV- Ca^2+^/calmodulin-dependent protein kinase II (CamKII)-GFP virions, which express GFP in dendritic compartments, including the shafts and spines, we observed a marked colocalization with endogenous CB1 in dendritic shafts and in several, but not all, dendritic spines (arrowheads in examples shown in Fig 6C). To morphologically pinpoint the localization of endogenous CB1 in synaptic spines, we then costained for the classical postsynaptic density protein, PSD-95, and observed a colocalization in a large number, but not all, PSD-95-positive spines (arrowheads in Fig 6C).

Similar results were obtained with exogenously-transfected CB1-EGFP (Fig 6D), which showed a marked colocalization with MAP2-positive dendrites in addition to being targeted to thin, MAP2-negative axons (Fig 6D).

Finally, we tested primary spinal cord neurons that were matured over 4 wk in culture and observed identical results—endogenous CB1 was markedly localized to MAP2-positive dendrites (arrows in Fig 6E) in addition to being expressed in MAP2-negative axons (arrowheads in Fig 6E).

### Cannabinoid Agonists Destabilize F-Actin in Dendritic Spines in Mature Neurons and Specifically Decrease Density of Mature Spines

**Fig 7:**
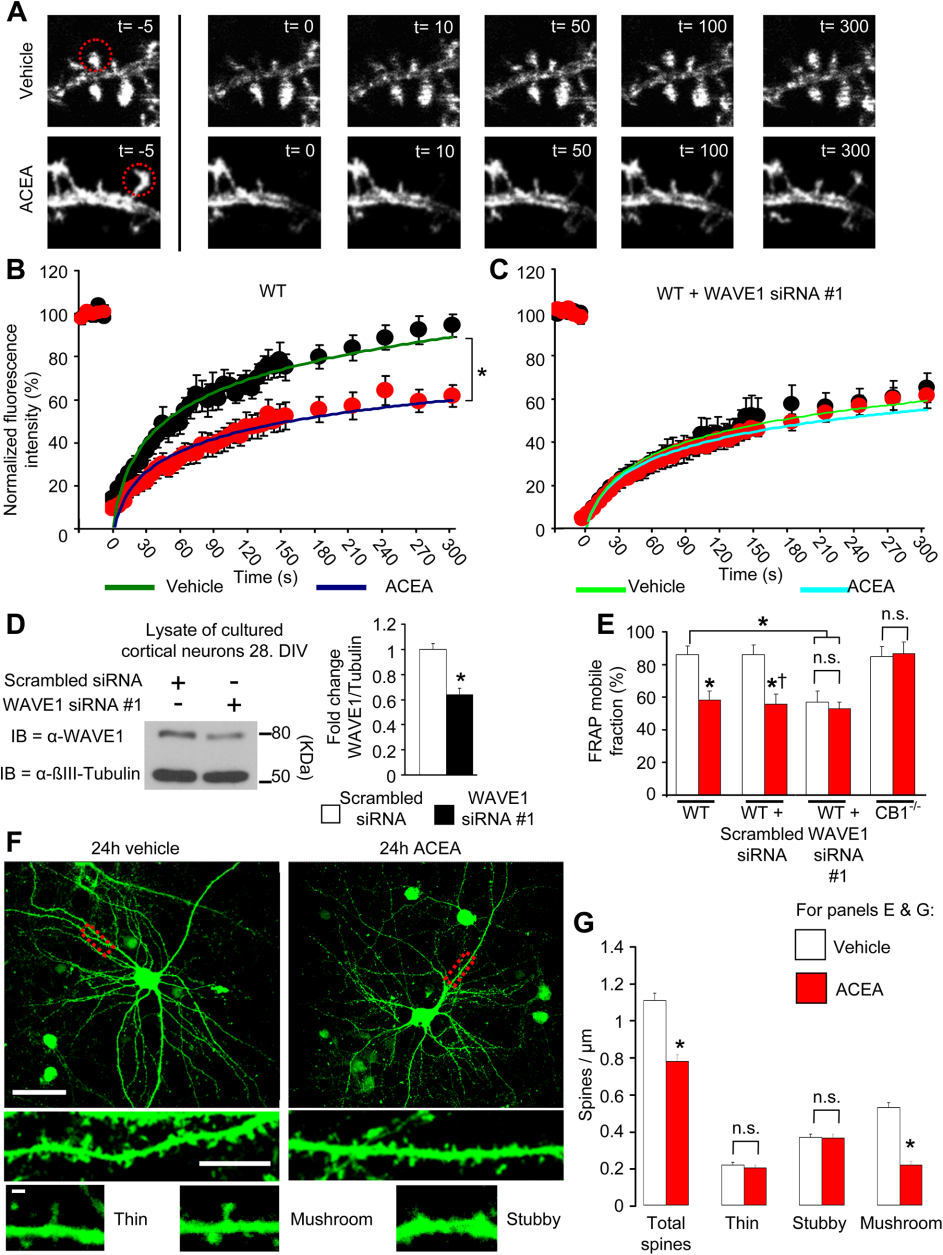
Visualization of disassembly of F-actin by cannabinoid agonists in in dendritic spines on mature cortical neurons, leading to reduction in the density of mature spines. (A) Representative real time images of FRAP experiments. Localized photobleaching over single, individual postsynaptic spines (red circles), led to loss of LifeAct-mCherry labeling of F-actin and progressive recovery of fluorescence with actin polymerization, which was inhibited by ACEA as compared to vehicle control. (B & C) Summary of FRAP values as a function of time expressed as one phase exponential function curves fitted to the respective data sets from neurons treated with ACEA or vehicle either from wild-type cultured cortical neurons (B) or with siRNA-mediated WAVE1 knockdown (C) (13–15 dendritic spines/group from 4 independent culture experiments). (D) Immunoblot example of WAVE1 down-regulation 3 d following siRNA delivery in mature cultured cortical neurons and the corresponding quantification (seven independent culture experiments). (E) Quantitative summary of the magnitude of F-actin recovery in the dendritic spine after photobleaching in neurons from the diverse treatment groups (five independent culture experiments). (F) Representative confocal images of mature cortical neurons transduced with rAAV-CamK-II-GFP virions and treated with ACEA (100 nM) or vehicle for 24 h. GFP-labelled dendritic spines were morphologically classified (lower panels) and quantified. Yellow bars represent 50 μm and white bars represent 5 μm. (G) Quantitative summary of average density of dendritic spines of varying morphology in cortical neurons treated with ACEA (100 nM) or vehicle (*n* = 20–21 dendrites analyzed from three cultures/group). All graphs represent mean values ± SEM *p < 0.05 as compared to control group and †*p* < 0.05 as compared to WT neurons, two-way ANOVA (E) or one-way ANOVA (D & G) followed by posthoc Tukey’s test. N.s. stands for “not significant.”

To address whether cannabinoids also regulate actin dynamics and thereby bring about structural remodeling in adult neurons via the WAVE1 complex, we then studied dendritic spines in wild-type cortical neurons matured over 3 wk in vitro and nucleofected with LifeAct-mCherry, thereby labelling F-actin in dendritic spines. To address dynamic changes in actin polymerization, we performed photobleaching of F-actin-bound LifeAct-mCherry in identified spines by focusing a light beam over individual spines and thereafter recorded the rate of fluorescence recovery after photobleaching (FRAP) [42] (image examples are shown in Fig 7A). Since LifeAct is known for its relatively low binding affinity for F-actin, this method may introduce indirect measures of F-actin turnover. We therefore restricted our quantitative analyses to the total recovery of the fluorescence mobile fraction of LifeAct-mCherry, which is indicative of the newly formed F-actin itself, at steady-state 150 s postphotobleaching. Quantitative analyses from five independent experiments revealed that the total recovery of the fluorescence mobile fraction of LifeAct-mCherry postphotobleaching was significantly lower in ACEA-treated neurons as compared to vehicle-treated neurons (Fig 7B and Fig 7E).

To test the relevance of WAVE1 for cannabinoid-induced modulation of F-actin assembly, we had to establish a method for knocking down WAVE1 in cortical cultures that had been maintained for several weeks in culture to develop synaptic spines (see Materials and Methods, example and quantitative summary of knockdown as compared to scrambled control shown in Fig 7D). FRET measurements on spines were performed 3 d after siRNA transfection, and WAVE1 knockdown but not scrambled siRNA blocked ACEA-induced remodelling of the actin cytoskeleton in dendritic spines (Fig 7C, Fig 7E and S5A Fig). Similarly ACEA failed to affect F-actin assembly in individual spines of primary cortical neurons derived from CB1^-/-^ mice (Fig 7E and S5B Fig). Thus, ACEA-modulated actin assembly in single postsynaptic spines of mature cortical neurons in a CB1 and WAVE1-dependent manner.

Because dendritic spines undergo considerable turnover and actin polymerization in spines is required for spine stability, formation, and development [43], the above results would predict that over time, activation of CB1 receptor results in a net reduction in spine density. To test this prediction, we transduced cultured cortical neurons with rAAV-expressing GFP under promoter elements of the mouse *CaMKII* gene (rAAV-CaMKII-GFP), thereby enabling visualization of dendritic spines on excitatory neurons 3–4 wk following culture and rAAV delivery (examples in Fig 7E). Neurons were treated with ACEA (100 nM) or vehicle (DMSO, 1:30,000 in phosphate-buffered saline [PBS]) for 24 h, and GFP-labelled dendritic spines on secondary and tertiary dendrites were imaged via confocal microscopy and morphologically categorized into “thin”-, “stubby”-, and “mushroom”-shaped spines as described previously [44]. We observed a net, significant reduction in dendritic spine density in neurons treated with ACEA as compared to vehicle (Fig 7G). Interestingly, morphological classification indicated that this change came about only with mushroom-shaped spines (Fig 7G), which are considered to represent stable and mature spines [44].

These results show that activation of CB1 dynamically modulates F-actin assembly in dendritic spines in mature cortical neurons and suggests that the net content of mature spines in excitatory neurons decreases over prolonged exposure to cannabinoidergic agonists.

### CB1 Receptor Agonists Inhibit Activity-Induced Remodeling of Synaptic Spines In Vivo via WAVE1

With a view towards testing the significance of these processes at the level of the whole organism, we sought a model system that would enable linking structural changes in spines in vivo with a functional change (e.g., in behavior) and also enable addressing the contribution of WAVE1 thereof using molecular interventions. We chose neurons located in the spinal dorsal horn in adult mice, owing not only to their involvement in the analgesic effects of cannabinoids but also because they undergo significant synaptic structural remodeling in inflammatory pain [45].

**Fig 8:**
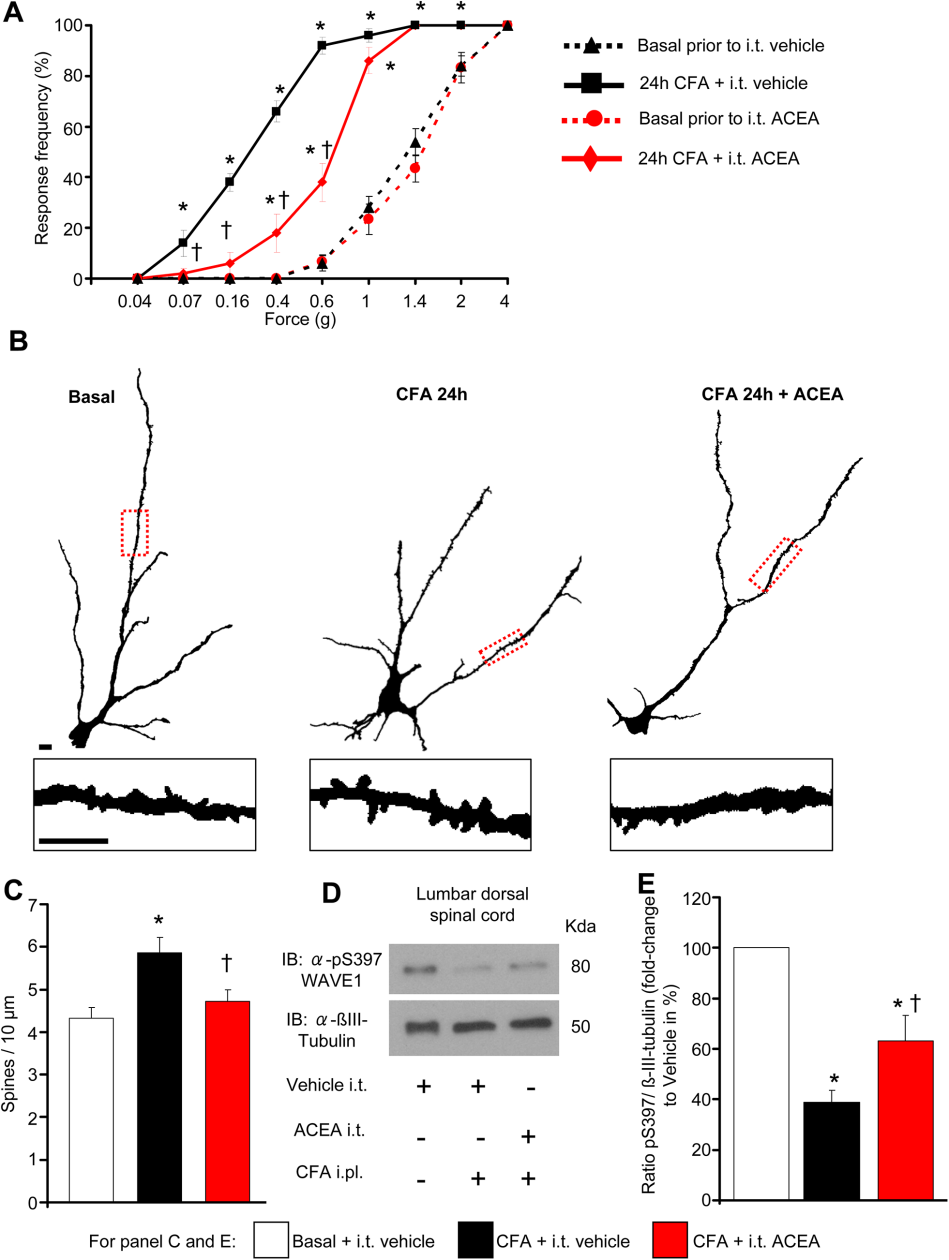
Intrathecal delivery of ACEA inhibits mechanical nociceptive hypersensitivity and nociceptive activity-induced structural plasticity of dendrites on spinal dorsal horn neurons in inflammatory pain conditions in vivo. (A) Frequency of paw withdrawal responses to mechanical force via plantar application of graded von Frey hairs recorded prior to and 24 h after hind paw intraplantar injection of CFA. Leftward shift in response curves following CFA (indicative of hypersenstivity) is diminished in mice treated intrathecally with ACEA (2 pmol) over 24 h as compared to mice intrathecally receiving vehicle (*n* = at least 8 per group). (B) Traced images of Golgi-stained large pyramidal-like neurons in laminae II and V of the spinal dorsal horn and dendritic spines (insets) in mice represented in (A). Scale bars represent 10 μm in all panels. (C) Quantitative summary of dendritic spine density in spinal neurons from mice represented in (A) and (B); CFA-induced enhancement in spine density does not occur in mice receiving intrathecal ACEA (*n* = 12–16 neurons counted from over 4 mice per group). (D, E) Mice with hind paw inflammation show reduced levels of pSer397 WAVE1, quantified over βIII-tubulin as loading control, in spinal lumbar segments L3-L5 at 24 h post-CFA, which is partially and significantly reversed by intrathecal ACEA as compared to vehicle. An example of western blot analysis and quantitative summary from seven mice per group is shown in (D) and (E), respectively. All graphs represent mean values ± SEM *p < 0.05 as compared to basal values within the group and †*p* < 0.05 as compared to corresponding values in the vehicle group, two-way (A) and one-way (C, E) ANOVA for repeated measures followed by posthoc Tukey’s test.

We employed a model of inflammatory pain based upon unilateral injection of complete Freund’s adjuvant (CFA) to the plantar surface of mouse hind paw, in which hypersensitivity to mechanical stimulation of the hind paw with von Frey filaments develops within 24 h of CFA injection (see leftward shift from basal values in the stimulus intensity-response frequency curves shown in black color Fig 8A). Consistent with previous reports of analgesic actions of spinally-applied cannabinoids [6], we observed that intrathecal delivery of ACEA to the lumbar spinal segments over the 24 h period post-CFA injection (three doses of 2 pmol ACEA given every 12 h) significantly attenuated inflammatory hyperalgesia (red-colored stimulus-response curves are shown in Fig 8A).

In ensuing morphological analyses of the spinal cords of mice analyzed behaviorally in the above experiment, we observed that the above-described functional changes were well-aligned with corresponding alterations in synaptic spine density on spinal neurons. Indeed, the development of inflammatory mechanical hypersensitivity in the CFA model is known to be associated with enhanced density of dendritic spines on neurons of the spinal laminae II and V [45], which play a key role in spinal nociceptive processing [46]. Likewise, in Golgi-staining experiments, we observed that CFA-injected mice treated intrathecally with vehicle showed increased spine density on the secondary or tertiary dendrites of spinal neurons in laminae II and V; however, spine remodeling did not occur in spinal neurons of mice treated over 24 h with ACEA (typical examples in Fig 8B and quantitative summary from 12–16 neurons from at least four mice shown in Fig 8C; these mice represent the same cohort of mice which were analyzed in behavioral studies shown in Fig 8A). Thus, spinal CB1 receptor activation suppressed nociceptive activity-induced dendritic spine remodeling in the spinal cord and inflammatory hypersensitivity in the same set of mice in vivo.

This gives rise to the question whether structural modulation by cannabinoids involves the WAVE1 complex. WAVE1 is ubiquitously expressed from E9, and the expression is restricted to CNS (including spinal cord), starting E15 until adulthood [47]. There are no reports on the potential contributions of WAVE1 to nociceptive modulation. Similar to our analyses on cortical neurons (Fig 3E to Fig 3G), we observed that WAVE1 shows serine phosphorylation in the basal state in the lumbar spinal cord segments involved in nociceptive processing in adult mice. Representative example of western blot analysis of pS397-WAVE1 and β-tubulin (loading control) are shown in Fig 8D and the corresponding western blot analysis of total WAVE1 expression is shown in S6A Fig. Quantitative summary for pS397-WAVE1 levels expressed as a function of either βIII-tubulin levels or total WAVE1 levels is shown in Fig 8E and S6B Fig, respectively. Our results indicate that activity of the WAVE1 complex in the spinal cord is limited under basal conditions. Interestingly, at 24 h following intraplantar hind paw injection of CFA in mice, the relative proportion of pSer397 WAVE1 decreased significantly, indicating an increase in WAVE1 activity over the period of initiation and expression of inflammatory hypersensitivity (Fig 8D, Fig 8E and S6B Fig). In mice receiving intrathecal ACEA over 24 h, CFA-induced decrease in pSer397-WAVE1 still occurred, but to a significantly lower magnitude than CFA-injected mice receiving intrathecal vehicle (Fig 8D, Fig 8E and S6B Fig). Thus, in parallel to decreasing nociceptive activity-induced spine remodeling in the spinal dorsal horn, ACEA-attenuated nociceptive activity-induced spinal activation of WAVE1.

**Fig 9:**
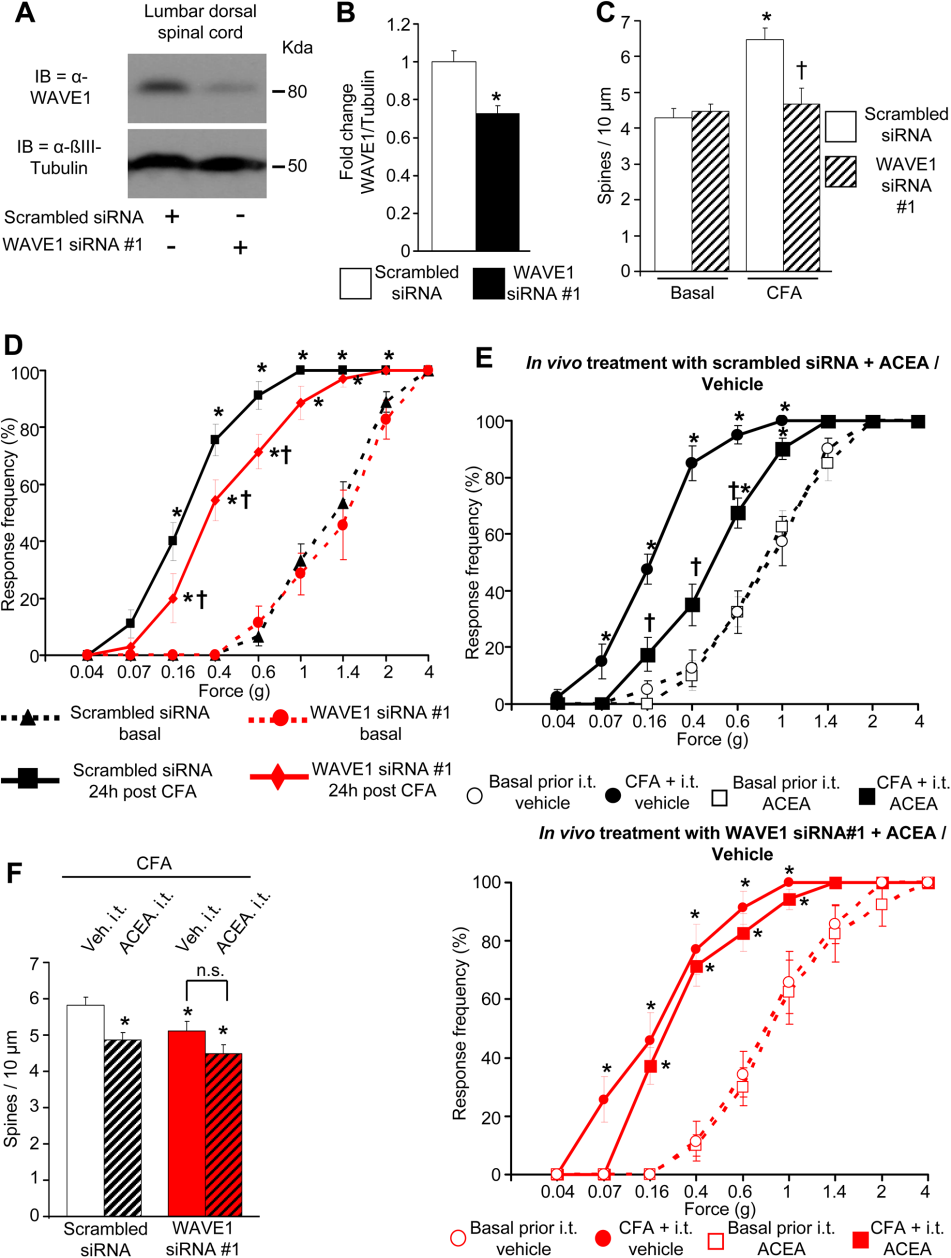
Role of spinally-expressed WAVE1 in nociceptive activity-induced structural plasticity, inflammatory pain, and cannabinoidergic analgesia in vivo. (A, B) An immunoblot example and quantitative data from western blot analysis showing down-regulation of WAVE1 in spinal cord segments L3–L5 after intrathecal in vivo application of WAVE1 siRNA as compared to control siRNA. (C) Normal basal spine density but lack of CFA-induced spine remodeling in lamina II/V lumbar spinal neurons at 24 h post-CFA in mice intrathecally injected with WAVE1 siRNA as compared to control siRNA (*n* = 16–17 spines counted over four mice per group). (D) Frequency of paw withdrawal responses to mechanical force via plantar application of graded von Frey hairs recorded prior to (dashed lines) and 24 h after hind paw intraplantar injection of CFA (solid lines). Leftward shift in response curves following CFA (indicative of hypersensitivity) is diminished with intrathecal siRNA-mediated WAVE1 knockdown (red symbols) as compared to mice intrathecally injected with control siRNA (black symbols). (E) Mechanical hypersensitivity at 24 h post-CFA is significantly reduced by intrathecal ACEA injection over 24 h in mice intrathecally treated with control siRNA (black squares in E, upper graph), but not in mice intrathecally treated with WAVE1 siRNA (red squares in E, lower graph). (F) Quantitative summary of dendritic spine density in laminae II or V neurons in L3–L5 segments of mice which were tested behaviorally in panels animals in (E). Intrathecal ACEA reduces spine density in control siRNA-injected CFA-inflamed mice, but not in WAVE1 siRNA-treated CFA-inflamed mice (16–18 neurons counted from over four mice per group). All graphs represent mean values ± SEM, *n* = 7–9 mice per group in panels D & E. *p < 0.05 as compared to basal values within the group and †*p* < 0.05 as compared to corresponding control, two-way (D, E) and one-way (B, C and F) ANOVA for repeated measures followed by posthoc Tukey’s test.

To directly test a potential causal relationship between spine remodeling and WAVE1, we performed similar experiments employing the CFA model in mice in which the spinal expression of WAVE1 was down-regulated by RNA interference in vivo. Inducing a partial knockdown of WAVE1 to about 70% of basal value (Fig 9A and Fig 9B) was preferable in the present experiments because it is known from previous studies that total knockout or a more substantial knockdown of WAVE1 decreases spine density in CNS neurons in conditions of basal activity [48]. Indeed, we observed that under basal conditions, the density of dendritic spines on laminae II/V spinal neurons was comparable in mice injected intrathecally with WAVE1-siRNA and scrambled-siRNA (Fig 9C). Consistent with the above, WAVE1-siRNA and scrambled-siRNA-injected mice showed comparable basal sensitivity to mechanical hind paw stimulation prior to induction of inflammation (stimulus intensity-response frequency curves represented by dashed lines in Fig 9D).

Importantly, unlike scrambled-siRNA-injected mice, WAVE1-siRNA-injected mice did not show enhancement of spine density at 24 h after intraplantar hind paw CFA injection (Fig 9C). In the same group of mice, the development of inflammatory hypersensitivity (represented by solid lines in Fig 9D) was partially, but significantly, attenuated in WAVE1-siRNA-injected mice as compared to scrambled-siRNA-injected mice (Fig 9D). Thus, a partial knockdown of WAVE1 expression in the lumbar spinal cord attenuated inflammatory hypersensitivity and abrogated activity-dependent spine remodeling in spinal dorsal horn neurons.

Finally, we asked whether modulation of WAVE1 mechanistically contributes to cannabinoidergic analgesia in the inflammatory pain setting. In scrambled siRNA-expressing mice, intrathecal administration of ACEA over 24 h following injection of CFA in the hind paw decreased the development of inflammatory hypersensitivity as compared to vehicle-treated mice (see solid lines in Fig 9E, upper graph) in a manner similar to our results from naïve mice described in Fig 8A above. However, in WAVE1 siRNA-expressing mice, ACEA did not significantly attenuate inflammatory hypersensitivity as compared to vehicle administration (see solid lines Fig 9E, lower graph). Dashed lines in both graphs in Fig 9E represent basal mechanical sensitivity in each of the respective groups prior to the induction of hind paw inflammation via CFA injection. In line with these behavioral observations, the above-described scrambled siRNA-injected mice demonstrated a decrease in CFA-induced spine remodeling in spinal neurons upon ACEA administration in comparison with vehicle (Fig 9F), similar to results obtained in naïve mice (Fig 8C). In contrast, loss of analgesic action of ACEA in mice with spinal WAVE1 knockdown was accompanied by failure of ACEA to attenuate CFA-induced spine remodeling in WAVE-siRNA-injected mice (Fig 9F). It is to be noted, however, that a loss of CFA-induced spine remodeling, which is already inherent to the WAVE1-siRNA-injected group of mice, may have led to a ceiling effect.

Taken together, these in vivo experiments indicate a requirement for spinal WAVE1 for the analgesic actions of spinally-delivered cannabinoids in inflammatory pain and reveal a close link between cannabinoidergic modulation of structural plasticity in spines and cannabinoid-induced analgesia.

## Discussion

This study uncovers new signalling mechanisms downstream of the CB1 receptor activation by identifying four components of the WAVE1 complex and Rac1 as physical interactors of the CB1 receptor. We elucidated the functional significance of this protein assembly in two key model systems, which have been critically associated with biological functions of the WAVE1 complex, namely growth cones of developing neurons and synaptic spine turnover in mature neurons.

Our results indicate that a physical association between Rac1-WAVE1 and CB1 receptor provides a direct molecular link between the CB1 receptor and actin rearrangement. This newly identified interaction gives mechanistic insights not only into known functions of cannabinoid agonists in the structural modulation of developing neurons but also provides evidence for a role for cannabinoids in modulation of actin assembly in dendritic spines on mature neurons of the CNS. This function is evident in diverse CB1 receptor-expressing neuron populations as demonstrated in developing as well as mature cortical neurons ex vivo and in spinal dorsal horn neurons in vivo. The importance at the whole organismal level is indicated by our finding that cannabinoid agonists suppress nociceptive activity-induced structural plasticity of synaptic spines in conjunction with their analgesic actions in inflammatory pain in a WAVE1-dependent manner.

The CB1 receptor complex has remained elusive, although a few interacting proteins have been described based upon candidate approach [18, 49]. This study reports an open, unbiased proteomic screen for CB1 receptor-interacting proteins in the mouse brain. Owing to their multiple transmembrane domains and structural peculiarities, GPCRs are notoriously problematic in immunoprecipitation analyses [50]. Our strategy of stereotactically expressing EGFP-tagged CB1 receptor via rAAV vectors coupled with GFP-Nanotrap on cortical membranes and LC-MS analyses enabled overcoming technical hurdles; given the propensity of GPCRs, including CB1 receptor, to heteromerize, our strategy pulled down CB1 receptor heteromers as well as several known protein interactors of CB1 receptor, such as the CB1 receptor-interacting protein CRIP1a [20], thereby validating the strategy and finding novel members of the CB1 receptor signaling complex.

Given our observation that the CB1 receptor also interacts with Rac1 and modulates its activity, cannabinoidergic modulation of WAVE1 activity may occur downstream of Rac1 since the WAVE1 complex is one of the most important effectors of Rac1 in bringing about actin remodelling [30]. We also additionally observed a bidirectional change in serine phosphorylation status of WAVE1 upon agonism and inverse agonism at the CB1 receptor in neurons, which, though modest in magnitude, was consistent and specifically mediated by CB1-Gi signalling. The WAVE1 complex has been suggested to act as a convergence point for integrating signals from diverse pathways that link extracellular signals as well as cell-autonomous programs to the modulation of the actin cytoskeleton [48]. cAMP signalling can affect WAVE1 activity via cyclin-dependent kinase 5 (CDK5)-mediated serine phosphorylation [36]. However, whether the cAMP-CDK5 pathway is interlinked with Rac1-mediated WAVE1 regulation or whether these run in parallel and represent independent modes of regulation of WAVE1 activity is not understood. Moreover, a recent study suggests that WAVE1 carries a highly conserved binding peptide motif, the WAVE regulatory complex (WRC)-interacting receptor sequence, which can facilitate interactions with diverse types of receptors, including some GPCRs [51]. However, we found that this motif is not conserved in the CB1 receptor. Given our observation that apart from WAVE1, three of the four remaining components of the WAVE1 signalling complex were coimmunoprecipitated with the CB1 receptor, it is plausible that any of them interact directly with the CB1 receptor and thereby mediate cannabinoidergic modulation of WAVE1 indirectly. The CB1 receptor may thus regulate WAVE1 serine phosphorylation as well as Rac1-mediated WAVE1 activation via steric modulation. Alternatively, CB1 receptor interactions with members of the WAVE1 complex may position WAVE1 optimally in its vicinity to facilitate regulation of WAVE1 activity by signalling mediators downstream of the activated G-protein. In this regard, it is interesting to note that cannabinoidergic modulation of WAVE1 activity, as well as downstream cellular effects thereof, were sensitive to Gi inhibition. Importantly, our results on the close colocalization between WAVE1 and CB1 in COS7 cells and in neurons, and importantly showing redistribution of WAVE1 to the cell membrane in cells treated with a CB1 agonist point to a key physical and functional link between CB1 and WAVE1. This was further strengthened by our observations that all of the effects of cannabinoidergic modulation on the actin cytoskeleton as well as morphology of neurons in vitro and in vivo as well as inflammatory pain behaviour were disrupted upon manipulating WAVE1 expression.

Another interesting observation was that agonists and inverse agonists acting via CB1 receptor bidirectionally altered the basal activation status of Rac1 and WAVE1 and, consequently, bidirectionally modulated actin nucleation, thereby implicating a considerable basal tone of activity of the CB1 receptor. It cannot be ruled out that this occurs in a ligand-dependent manner, because despite the absence of serum and supplements during assays, traces of ligands may persist in the medium. On the other hand, there is rising evidence for ligand-independent constitutive activity of a number of GPCRs, including CB1 receptor [40,52]. The constitutively active "on" state of CB1 receptor can be shifted reversibly into constitutively inactive "off" state through conformational changes of the receptor, and thus bidirectionally modulate Rac1 and components of the WAVE1 complex, leading to changes in WAVE1 activity. In this two-state model, the existence of ligands, either agonist or inverse-agonist, would shift the proportion of the receptors to the active or inactive conformation [52,53]. Irrespective of which of the above scenarios comes into play, the observation that tuning CB1 receptor activity up or down profoundly influences the Rac1-WAVE1-actin nucleation axis places the CB1 receptor in a key position to sense diverse endocannabinoids and lipid modulators of CB1 receptor and bring about localized regulation of actin dynamics.

How actin dynamics are affected by cannabinoids in developing neurons is not mechanistically well understood. Here, direct visualization of F-actin in growth cones revealed that cannabinoid agonists destabilize F-actin in growth cones, whereas deactivation of CB1 receptor activity by inverse agonists leads to enhanced actin nucleation in growth cones. This cannabinoidergic modulation of actin dynamics was entirely in line with the changes in morphology of axonal growth cones brought about by CB1 receptor agonists and inverse agonists in developing cortical neurons. Interestingly, a very recent study has reported a role for endocannabinoid in stabilizing microtubules via degradation of SCG10/stathmin-2 protein in developing neurons [54], which is complementary to our results on actin remodeling. Taken together, these findings indicate a prominent role of the CB1 receptor in cytoskeletal rearrangement.

Most analyses of RhoGTPase modulation are carried out via pull downs on cell lysates thereby representing net RhoGTPase activity over diverse cells as well as diverse compartments of the cell. Imaging studies, however, have revealed that within a cell, RhoGTPase activity varies substantially in a highly compartmentalized manner, e.g., between the leading edge and the cell interior. Here, direct visualization of localized Rac1 activity within the growth cone revealed a decrease in Rac1 activity within minutes of CB1 receptor activation, whereas total Rac1 in somata did not change considerably. This, taken together with the observation that inverse agonism at the CB1 receptor conversely increased Rac1 activity, suggests that a basal tone of CB1 receptor signaling controls basal Rac1 via protein–protein interactions in growth cones of cortical neurons, which can be dynamically tuned to (endo)cannabinoid content in the environment. Given that RhoA and Rac1 exert opposing effects on actin polymerization, by activating RhoA [12] and deactivating Rac1 (present study), agonists at CB1 receptor would be optimally positioned to bring about acute, localized disassembly of F-actin, leading to collapse of growth cones. Moreover, the two RhoGTPases likely act in differential subcellular domains, with RhoA highly functional in the periphery of lamellipodial segments of the growth cone, and Rac1-WAVE1 being expressed throughout the growth cone bodies, especially in the tips and shafts of filopodia [55,56], which is consistent with our observations of RaichuRac activity in FRET imaging experiments, thereby leading to concerted filopodial and lamellipodial retraction upon CB1 receptor activation. Conversely, our results showed that CB1 receptor inverse agonists stabilize the F-actin assembly in growth cones concurrently to Rac1 activation. This suggests that an acute decrease in the local tone of cannabinoidergic signaling would enable growth cones to temporarily stabilize and enlarge, a step which is important not only in axonal navigation, but also facilitates branching in axons [57].

Importantly, the data indicate that cannabinoidergic modulation of actin remodelling via WAVE1 occurs in developing as well as mature neurons and spans distinct systems, such as the cortex and the spinal cord. Furthermore, we found strong evidence for localization of CB1 in axonal as well as dendritic compartments in mature neurons, including in postsynaptic spines. The highly restricted nature of our manipulation of actin assembly in individual spines in FRAP experiments, which was rapidly altered upon cannabinoidergic modulation, coupled with the evidence for CB1 localization in dendritic spines makes it likely that the functional effects evoked by cannabinoids in this study indeed result from CB1 activation in dendritic spines. However, we cannot rule out that previously described presynaptic functions of CB1, such as modulation of neurotransmitter release from a presynaptic locus, play a role in the effects observed here. Our observation on cannabinoidergic modulation of dendritic spines is particularly interesting given that these sites of synaptic transfer are highly motile structures that govern key functions and act as important sculptors of plasticity in the CNS. Here, direct visualization of actin assembly via FRAP experiments in adult cortical neurons revealed that CB1 receptor activation significantly limits the conversion of G-actin to F-actin, leading over time to a depletion of mature spines with an elaborate mushroom morphology, which are believed to mediate synaptic stability and increased synaptic efficacy such as long-term potentiation (LTP) [44].

Moreover, these results also expand our understanding of the therapeutic effects of cannabinoids and clarify mechanistic contributions. Here, we utilized spinally-mediated analgesic functions of CB1 receptor agonists as another model system to test the impact of CB1-WAVE1 interactions on structural and functional plasticity at the level of the whole organism. We observed that intrathecal delivery of the CB1 receptor-specific agonist ACEA countered inflammatory mechanical hypersensitivity when given prior to the inflammatory insult, i.e., before the onset of pathological nociceptive activity. That this functional modulation is associated with structural remodeling was revealed by the observation that parallel to attenuation of inflammatory hypersensitivity behavior, ACEA also decreased nociceptive activity-induced spine remodeling in spinal neurons, a structural correlate for spinal potentiation and central sensitization.

Interestingly, the results of this study also describe a novel function for WAVE1 in the modulation of pain, particularly in mechanisms of nociceptive sensitization. Since spinal dendritic spine remodeling has been suggested to underlie diverse pathological pain states, including neuropathic pain associated with nerve lesions, diabetes, and HIV neuropathy [58], it will be interesting to address in future studies whether WAVE1 mechanistically contributes to underlying processes and whether spinal cannabinoids may constitute an effective therapy.

Despite its breadth spanning diverse ex vivo and in vivo analyses and model systems, this study has several limitations. First, given that the WAVE1 complex is large and has numerous protein components that coassemble with the CB1 receptor, the precise nature, order, and molecular structure of the interactions has not been worked out and will constitute a large study in their own merit. Furthermore, it is important to note that the expression of the CB1 receptor is not restricted to the excitatory neurons, which were mainly addressed in this study, but is highly pronounced in axons of GABAergic neurons [59]. It remains to be addressed whether CB1-WAVE1 interactions play a role in cannabinoidergic modulation of interneurons. Finally, WAVE1 may have additional downstream targets apart from actin nucleation, such as kinesin-1, CRMP-2 and profiling [30,60], and it remains to be determined whether and how these are related to cannabinoidergic mechanisms.

In summary, our findings identify cannabinoids as key upstream regulators of the WAVE1 complex via physical interactions within the CB1 receptor assembly, which play a fundamental role in actin dynamics and structural modulation in growth cones during development, as well as in activity-induced plasticity of dendritic spines in adult life. This study uncovers novel mechanisms for developmental functions as well as therapeutic applications of cannabinoids and implicates the WAVE1 complex as a mediator of plasticity processes leading up to inflammatory pain.

## Materials and Methods

### Animals

All animal experiments were performed in accordance with the EU guidelines 2010/63/EU and the German TierSchG and TierSchVersV. All animal experimental protocols were approved by the local governing body Regierungspräsidium Karlsruhe (license numbers T43/13, T49/14, G86/11 and G192/14) and were performed in accordance with their ethical guidelines. All in vivo experiments were done in age-matched (8 wk) male C57Bl6 mice. Conventional CB1 global knockout mice (CB1^-/-^) have been described previously [32].

### Virus Production and In Vivo Injection

A rAAV vector with an AAV1/AAV2 chimeric backbone was used to express GFP or CB1 tagged with EGFP on its N-terminal end [22]. Viral particles were produced using AAV-293 HEK cell line (Stratagene), a derivation of HEK-293 that produces higher viral titres. The cells were transfected with equimolar amounts of pAM-EGFP-tagged CB1 or pAM-GFP plasmid, pDP1rs helper plasmid and pDP2rs helper plasmid (PlasmidFactory) that were previously mixed in a transfection buffer (140 nM NaCl, 25 mM HEPES, 750 μM Na_2_HPO_4_, and 125 mM CaCl_2_, pH 7.05). Three days following transfection, the cells were lysed with a lysis buffer (150 mM NaCl, 50mM Tris-HCL pH 8.5) to harvest the viral particles. To purify the viral particles, the attained lysate were loaded into a pre-equilibrated heparin agarose column, incubated for 1–2 h and washed at least four times with equilibration buffer (1x PBS, 1 mM MgCl_2_, 2.5 mM KCl, pH 7.2). The viral particles were then eluted out of the column with elution buffer (equilibration buffer with 0.5 NaCl and pH 7.2) and then washed and centrifuged in Amicon-ULTRA filter to get rid of the salt and concentrate the particles. Following harvest, the viral titre was determined, and brain injection of virions (2–5 * 10^9^ particles/ml, 500 nl) in C57Bl6 adult mice were performed stereotactically as previously described [22]. Briefly, C57Bl6 adult mouse at 8–10 wk of age were anesthetized with fentanyl/medetomidine/midazolam (4:6:16; 0.7 μl/g, i.p.), and after the surgical anesthesia is reached (lack of response to noxious stimuli), the fur on the head was shaved and cleaned with 70% ethanol. The head of the animal was then fixed on the Stereotaxic Alignment System (Kopf instrument, model 1900), leveled, and incised carefully along the midline with adequate local application of lidocaine during incision. Ten coordinate points were determined as such that these were distributed evenly across the brain with five points at each hemisphere by using the bregma as point [0,0]. Afterwards, craniotomy was performed carefully with handheld drill on determined points. Following this, injection of rAAV (injection depth 500 μm; injection volume 500 nl of the rAAV stock, diluted in PBS; 2–5 * 10^9^ particles/ml) into the exposed cortex was performed very slowly using a glass micropipette that was fixed into the holder of the stereotaxic arm (injection depth 500 μm). After injection, we waited another 2–3 min before withdrawing the glass micropipette carefully to avoid backflow of the virus into the surface. After all injections were done, the skull was cleaned with sterile PBS and sprayed once again with lidocaine solution, and the skin was sutured. Afterwards, the animal was let to recover, and we waited at least 4 wk to make sure that the recombinant in vivo expression from the AAV vector was sufficient before the mouse was used for further experiments.

### Membrane Protein Solubilisation and Immunoprecipitation

Cortex tissue or cultured cells were homogenized with bench homogenisator in ice-cold hypotonic buffer (20 mM Hepes pH 7.5, 5 mM EDTA, 10 mM DTT, and 1x protease inhibitor cocktail (Roche)). After a brief centrifugation at 800 G 4°C, the supernatant of the homogenate was further subjected to ultracentrifugation at 125.000 G 4°C for 45 min to concentrate membrane-bound proteins in the pellet. The pellet from this ultracentrifugation was then solubilized for at least 2 h, 4°C in solubilization buffer (1% (w/v) Na-cholate, 5 mM EDTA, 100 mM NaCl, 20 mM Hepes pH 7.5, 10 mM DTT and 1x protease inhibitor cocktail (Roche)). Subsequently, this solubilized lysate was ultracentrifuge at 100.000 G, 4°C for 45 min to separate the solubilized membrane protein (supernatant) from the unsolubilized fragments (pellet) [61].

Following solubilization, the lysates were further diluted with hypotonic buffer in 1:3 ratio. GFP-nanotrap beads [26] (Chromotek) or protein A/G PLUS agarose beads (Santa Cruz) coupled with rabbit-α-CB1 (Frontier Science) were then added into the solution and the mixture was incubated for at least 2 h or overnight (α-CB1-coupled beads) at 4°C in an overhead tumbler.

For GFP-nanotrap immunoprecipitation, the beads were collected and washed three times with ice-cold hypotonic buffer in Pierce Microcentrifuge Spin Column (Thermo Fischer). The beads were then resuspended in hypotonic buffer, mixed with 5 x Laemmli buffer in 4 to 1 ratio, and cooked at 95°C with vigorous shaking in a thermoblock for 5 min and was used for further analyses.

For conventional immunoprecipitation using protein A/G beads coupled to α-CB1, following overnight incubation with solubilized lysate, the beads were collected and washed at least five times with ice-cold washing buffer (20 mM Hepes pH 7.5, 5 mM EDTA, 10 mM DTT, 1x protease inhibitor cocktail, and 400nM NaCl). Subsequently, the beads were briefly washed with Elution Buffer (Thermo Scientific) once. The beads were then incubated with Elution Buffer for at least 1 min, following which the eluate was collected. 1M TRIS buffer pH 9.5 was added in the dilution of 1:20 to the eluate to neutralize the acidic Elution Buffer, mixed with 5 x Laemlli buffer, cooked at 95°C with vigorous shaking in a thermoblock for 5 min, and subjected to further analyses.

### MS

Immunoprecipitates were subjected to LC MS/MS analysis using a nanoHPLC system (Eksigent, Axel Semrau) coupled with an ESI LTQ Orbitrap mass spectrometer (Thermo Fisher). Attained MS/MS spectra results were searched against the swiss prot protein database, selected for *Mus musculus* (16,338 entries) using the Mascot software (Matrix Science). Five independent immunoprecipitates were subjected to MS analysis. Subsequently, the uninterpreted MS/MS spectra were searched against the swiss prot protein database, selected for *M*. *musculus* (16,338 entries) using the Mascot software (Matrix Science). The algorithm was set to use trypsin as proteolytic specificity, assuming carbamidomethyl as a fixed modification of cysteine, and oxidized methionine and deamidation of asparagines and glutamine as variable modifications. Mass tolerance was set to 100 ppm and 0.5 Da for MS and MS/MS, respectively. Only protein hits with a probability of *p* < 0.05 for a random match were listed [62].

MS data was evaluated using rPQ score [63]. rPQ score is the ratio of peptide queries obtained for any given protein from GFP-nanotrap immunoprecipitate of the rAAV-EGFP-tagged CB1-transduced brain and control (eluates of the rAAV-GFP transduced brain lysate). For proteins that were not detected in the controls, a detection limit of 0.125 (queries) was used as a denominator in the rPQ score. Proteins with rPQ values above four were regarded as specific.

### Primary Cell Culture

Cultured cortical neurons were prepared from C57Bl6 mouse embryos from embryonic day 16 (E16) as previously described [64] and transfected using nucleofection device (Lonza) and P3 Primary Cell 4D-Nucleofector Kit (Lonza) either with plasmids and/or siRNA according to the respective experiments.

For experiments on developing neurons, approximately 20,000 cells/cm^2^ were seeded on coverslips inside a 12-well plate (growth cone modulation assay) or 10 cm petri dish (WAVE1 phosphorylation assay). For experiments requiring mature cultured neurons, approximately 120,000 cells/cm^2^ were seeded on 14 mm or 24 mm diameter coverslips and cultured for 4 wk. The medium was changed 1 d after preparation with fresh B27-supplemented neurobasal medium (500 ml Neurobasal medium, 10 ml B27, 150 mM GlutaMax, penicillin/streptomycin 50 U/ml, 0.88 μl β-mercaptoethanol). For older culture, half of the medium was replaced every 6 d with a mixture of B27-medium and N2-supplemented (Life Technologies) neurobasal medium (500 ml Neurobasal medium, 5 ml N2, 150 mM GlutaMax, penicillin/streptomycin 50 U/ml, 0.88 μl β-mercaptoethanol) in ratio of 1:2.

To asses modulations of growth cones and WAVE1 phosphorylation, three days in vitro (DIV) cultured neurons were treated with different compounds: (1) DMSO (Calbiochem) (used as vehicle for all compounds, unless stated otherwise, diluted to 1:30,000 in neurobasal medium) (2) ACEA (Sigma), working end-concentration 100 nM or (3) AM251 (Sigma), working end-concentration 600 nM. Treatment lasted for 1 h (for growth cone assay) or 45 min (for WAVE1 phosphorylation assay). Subsequently, the cultured neurons washed with prewarmed PBS and swiftly fixed with ice-cold 4% paraformaldehyde (PFA, Sigma) for growth cone modulation assay. For WAVE1 phosphorylation assay, after washing with prewarmed PBS, the cultured neurons were then lysed with ice-cold 1x RIPA buffer and shaken in an overhead tumbler for 20 min at 4°C. Lysed neurons were then pelleted with centrifugation at 13.000 RPM at 4°C and the supernatant was collected, mixed with 5 x Laemmli buffer, boiled with vigorous shaking at 95°C for 5 min, and subjected to immunoblotting.

For G_i_-inhibition, cultured neurons were pretreated with 100 ng/ml PTX, diluted in water (Sigma) overnight (approximately 20 h) prior to the treatments with ligands. For inhibition of Rac1, neurons were pretreated with 50 μM Rac inhibitor II or Z62954982 (Calbiochem) for 4 h prior to treatment. WAVE1 inhibition was done by nuclecofecting cells WAVE1 siRNA before plating them.

To analyse and quantify dendritic spines, the neurons were transduced with rAAV-CaMKII-GFP at 7 DIV and cultured further for at least another 2 wk. The mature cultured neurons were then treated with either vehicle (DMSO 1:30,000) or ACEA (100 nM) for 24 h. Following fixation, neurons were imaged with laser-scanning confocal microscope (Leica SP2 AOBS) using a 100X objective (Leica). Only spines from 2nd order dendrites or higher were taken into account.

### WAVE1 Redistribution Assay in COS7 Cells

COS7 cells were cultured in complete DMEM (Gibco) medium on poly-D-lysine (Sigma) coated glass coverslips. After reaching sufficient confluency (approximately 60%–70%), cells were transfected with either GFP or CB1-EGFP together with mWAVE1 plasmid (OriGene) using Lipofectamine 2000 (Invitrogen). At least 24 h post transfection, the cells were treated either with DMSO (1:30,000) or with 100 nM ACEA for 45 min, washed with prewarmed PBS, fixed in ice-cold 4% PFA for 20 min, and washed twice with RT PBS before storing or used in immunostaining against WAVE1 and actin filaments.

Following immunostaining, cells that are either positive for GFP or CB1-EGFP were individually imaged using SP8 confocal microscopy (Leica) with the exact same conditions throughout. Captured images were then analyzed using Fiji software. A ROI of the cell membrane (membrane ROI) of each individual cell was created by creating a binary mask of the Phalloidin staining at the cell membrane. The ratio of WAVE1 intensity against the Phalloidin intensity at the corresponding membrane ROI was used to quantify the WAVE1-immunoreactivity at cell membrane.

A second ROI was created for WAVE1 and CB1-EGFP colocalization (colocalization ROI). To achieve this, the image of WAVE1 immunostaining of individual cell was overlapped to the corresponding image of CB1-EGFP using the “AND” command in the Fiji software. The ratio of CB1-EGFP total intensity at the colocalization ROI to the CB1-EGFP total intensity of whole cell was used as the quantification of the intensity of CB1-EGFP-WAVE1 overlap.

### Raichu-Rac1 FRET Imaging

Cultured cortical neurons were nucleofected with pRaichu-Rac1 cDNA plasmid (Prof. Michiyuki Matsuda) and plated with the density of approximately 20,000 cells/cm^2^ on 24 mm cover slips. The cultures were then starved stepwise by replacing half of the culture media with fresh nonsupplemented neurobasal medium twice on 2nd DIV and a couple hours prior to measurement which is done on 3rd DIV. For FRET measurement, the cover slips with starved cells were mounted on a cover slips holder filled with nonsupplemented F12 medium (Gibco). Cells were imaged with an inverted microscope (Nikon Ti-E, Nikon) with a Nikon 100x Plan Apo objective with NA 1.4. The microscope was equipped with a hardware autofocus (Nikon PFS) and a stage top incubation system (INU series WSKM, TOKAI HIT, Japan). The FRET sensor was excited with a metal halide light source (Intensilight, Nikon) gated by a 425/26 nm band path filter. The emission was separated by a two-camera adapter (TuCam, Andor Technology) with a beam splitter at 509 nm onto two scientific complementary metal oxide silicon (sCMOS) cameras (sNeo, Andor Technology)). The first camera was gated by a CFP filter (465/30 nm) and the second by a YFP filter (550/49 nm). All filters were of the hard-coated kind and purchased from AHF, Tübingen. Acquisition was driven by NIS-Elements Software 4.1 (Nikon) with simultaneous acquisition of CFP and YFP channel by hardware triggering between the cameras. For measurement of basal activity prior to any stimulation, time-lapse image series were acquired with 100 ms exposure time every 45 s for 15 min. After the basal measurement, the cells were treated with following pharmacological agents: DMSO (diluted to 1:30,000 in F12), ACEA (100 nM), AM251 (600 nM) and NGF (Sigma) (diluted in PBS, working end-concentration 100 ng/ml), and another image series were taken with the same conditions for 45 min.

Image processing was done using NIS-Elements Software 4.1. Channels were aligned by a linear transformation (rotation and shift). In both channels, the background was subtracted frame by frame by defining a background region of interest. For each frame, a ratio image YFP/CFP was calculated and represented as pseudocoloured intensity map, which relatively corresponds to the state of activated Rac1. Regions in the nuclear and growth cone areas were defined, and average ratios in these areas plotted against time. The raw ratios from individual experiments were divided by the average basal value to get the normalized values time traces.

### LifeAct Time-Lapse Imaging

Cultured cortical neurons were nucleofected either with LifeAct-GFP cDNA plasmid (Ibidi) only, or together with WAVE1 siRNA or scrambled siRNA (100 nM, SCBT) before plating them on 24 mm cover slips with the density of 20,000 cells/cm^2^. The following siRNAs were used for transfection:

1. WAVE1 siRNA #1 (a pool of 3 different duplexes, SCBT): CGGAGUUCGAUGAAGUAGA; GCCUUAUUCCGUUUCUUGA; and GGAACAUGUAGCUUGUAGU.
2. WAVE1 siRNA #2 (a pool of 3 different duplexes, OriGene): AGAUAUAACGAUGAGAAAGGCUU; GGUAUUCAGCUUCGCAAAGUGGA; and GCCUCGAUUAUAUGAAUACAC.
3. Scrambled siRNA MISSION siRNA Universal Negative Control #1 (Sigma-Aldrich).

On the 3rd DIV, time-lapse images and treatment were done in a similar fashion as the FRET imaging described above, but without beam splitter and only with one sCMOS camera. Moreover, LifeAct-GFP was excited with a metal halide light source gated by a 482/18 nm band path filter.

Image processing was done using Fiji. Briefly, images were converted into binary image, and a mask of the growth cone area was created. The masking was superimposed back into the original 16 bit image, and the area and intensity of the growth cone were measured. For each cell, measurements were taken from images at 0, 30th, and 60th minutes (for intensity additionally at 15th and 45th minutes). The raw values of each time point were normalized against the basal value at 0 minute and presented as percentile ratio.

### FRAP Imaging

Cultured cortical neurons were prepared as previously described and nucleofected with LifeAct-mCherry cDNA (Ibidi). After approximately 25 DIV, the matured cultured neurons were transfected either with WAVE1 siRNA #1 or scrambled siRNA (both at the concentration of 25 nM) using HiPerFect kit (Qiagen). Three days after siRNA transfection, the matured cultured neurons were imaged at 37°C using an inverted microscope (Nikon Ti-E, Nikon) coupled to A1R confocal system (Nikon) with an oil immersion objective (Nikon Plan Apo λ 60x NA 1.40). 561 nm Argon-laser was used to excite LifeAct-mCherry. Photobleaching of LifeAct-mCherry was acquired by constant laser exposure for 25 s and concentrating the exposure to a predefined circular (3 μm diameter) ROI that is targeted to individual spines. The laser power used to capture images and bleaching was set to 5% of the maximum laser capacity. To examine the recovery of LifeAct-mCherry after photobleaching on these spines, series of images were taken at 5 s interval for the first 2.5 min and further at 30 s interval for another 2.5 min.

Following background subtraction, the fluorescence intensity of target spines was normalized to the fluorescence intensity of a nearby unbleached “reference” spine. These normalized values were then compared to the average intensity of 5 images on basal state (prior to bleaching) to get the FRAP values. Using Prism software (GraphPad), these values were fitted to one-phase exponential function: y = y_0_ + a(1 − exp^(−Kx)^), where y_0_, a, and K are offset, maximum value and time constant, respectively. Mobile fraction was calculated from the average of the last five images and set as the maximum recovery value (y_0_ + a).

### Immunostaining

Immunohistochemistry and immunoblotting were done as described previously [45], with a minor change in blocking solutions. For immunostaining, blocking was done using PBS containing 3% IgG-free BSA (Jackson Immuno Research) and 4% normal horse serum. For immunoblotting immunoprecipitation lysate, blocking was done using PBST containing 3% BSA (Roth) and 3% nonfat Milk (Roth). Immunostaining and–blotting were done with following primary antibodies: polyclonal goat anti-CB1 (Prof. Ken Mackie); polyclonal rabbit anti-CYFIP2 (Sigma); polyclonal goat anti-pGAP43 (SCBT); monoclonal mouse anti-GFP (Neuromab); polyclonal rabbit anti-NCKAP1 (Sigma); monoclonal mouse anti-Rac1 (BD Bioscience); monoclonal mouse anti-Tau1 (Merck); polyclonal rabbit anti-βIII-Tubulin (Sigma); polyclonal goat anti-WAVE1 (R&D); polyclonal rabbit anti-WAVE1 (Sigma) polyclonal rabbit anti-pospho-WAVE1 (Sigma). Actin fibres were visualized with TRITC-conjugated Phalloidin (Invitrogen).

### Golgi Staining

Golgi–Cox staining was performed using an FD Rapid GolgiStain Kit (FD Neurotechnologies) according to the manual and as previously described [45]. Following impregnation steps, the tissues were shock-frozen in iso-pentane solution precooled in dry ice and afterwards cut with cryostat in sections with 90 μm thickness and mounted in gelatin-coated glass slides. The cut tissues were allowed to dry for at least overnight, then stained by immersing them in a mixture of staining solution from the kit, dehydrated in ethanol, cleared in xylene, and mounted using Eukitt (O. Kindle) mounting media.

For analysis purposes, only impregnated neurons with diameter of at least 20 μm that were located within the laminae II to V of the spinal dorsal horn were chosen. Moreover, only secondary or tertiary dendrites that were projected in the direction of the dorsal horn were further analyzed. Images were captured using an upright microscope (Nikon NiE, Nikon) equipped with Nikon Plan Apo objective set and high resolution CCD camera (Nikon DS-Ri1, Nikon). Acquisition process was driven by NIS-Elements software 4.1 (Nikon).

### Intrathecal Delivery

siRNAs were prepared as previously described [65]. Briefly, siRNA (for each mouse) was diluted with water up to a quarter of the desired end volume needed for intrathecal delivery and diluted further with 10% glucose solution up to half of the desired end volume. The PEI solution (Polyplus) was prepared in similar fashion, first dilution with water up to quarter of the end volume and then again with 10% glucose solution up to half of the end volume. The diluted siRNA solution was then mixed with the diluted PEI solution and incubated for at least 15 min at RT before injection. For down-regulation of WAVE1, 1.2 μg siRNA (scrambled siRNA or WAVE1 siRNA) (SCBT) was prepared in 15 μl-PEI mix solution for each mouse.

ACEA and DMSO were diluted in artificial cerebrospinal fluid (ACSF; 119 mM NaCl, 26.2 mM NaHCO_3_, 2.5 mM KCl, 1 mM NaH_2_PO_4_, 1.3 mM MgCl_2_, 10 mM Glucose, 2.5 mM CaCl_2_, pH 7.3). ACEA was diluted to reach the concentration of 200 nM, and DMSO was accordingly diluted 1:500,000 in ACSF. 10 μl of this diluted solution was used for every round of treatment.

### Somatic Inflammation Model

CFA Sigma was injected unilaterally in the intraplantar surface of the hind paw in mice (30 μl), whereas control mice were injected with 0.9% saline [66].

### Measurement of Nociceptive Sensitivity

Mechanical sensitivity was measured by applying punctuate pressure using Von Frey filaments (Ugo Basile) of different strength [66]. The filaments were applied perpendicularly to the plantar surface in the middle of the mouse’s hind paw with an upward force just sufficient to bend the microfilament. A positive response is a paw withdrawal before the filament bending, and we measured frequency of response out of five applications. Force required to elicit 40% response is considered mechanical thresholds. All behavioural measurements were done in awake, unrestrained, age-matched male mice by individuals who were blinded to the treatment of the mice being analyzed.

### Data and Statistical Analysis

All image analyses were done by experimentor that was blinded to the identity of the samples being analyzed. All data are expressed as mean ± SEM. Individual statistical analyses are stated in the respective figure legends. Changes with *p* < 0.05 were considered to be significant. No statistical methods were used to determine sample sizes.

## Supporting Information

S1 Data Raw numerical values for all quantitative analyses in the main and supplementary figures. Raw values from Fig 2B and 2C; Fig 3C, 3D and 3G; S3B–S3E Fig; Fig 4B–4I and 4K; S4B and S4C Fig; Fig 5B–5D; Fig 7B–7E and 7G; S5A and S5B Fig; Fig 8A, 8C and 8E; S6B Fig; Fig 9B–9F; S1 Table. Each figure is separated into different worksheet.
(XLS)

S1 Fig Control experiments to support immunoprecipitation of interaction partners of CB1 in mouse cortex. (A) Validation of bands recognized by the anti-CB1 antibody in membrane fractions derived from wild-type mice (WT) and mice lacking CB1 globally (CB1^-/-^ mice). Only the bands indicated by black arrowheads, constituting monomeric and multimeric CB1, are specific and the uppermost band corresponds to the multimeric CB1-EGFP band indicated by arrow and arrowhead in panel A. (B) Overview of known (published) interaction partners of CB1 found via LC-MS analysis on immunoprecipitates derived from mouse cortically expressing CB1-EGFP, but not in mice expressing EGFP alone. Shown are rPQ, with values above four indicating significant interactions.
(TIF)

S2 Fig Coimmunoprecipitation and coexpression analyses on CB1 and the WAVE1 signaling complex. (A) In HEK cells transfected heterologously with CB1-EGFP or with GFP, endogenous WAVE1 coimmunoprecipitated with CB-EGFP, but not with GFP alone. Expression controls for GFP and CB1-EGFP in transfected cells are shown on the right. (B) Pseudocolored images showing colocalization between endogenously expressed CB1, actin, and WAVE1 in immunostained cultured cortical neurons. Magnified image represent white box inset. White arrowheads in inset and magnified image point to the growth cone. Scale bars represent 20 μm.
(TIF)

S3 Fig Analysis of Rac1 activity based on FRET analysis in developing cortical neurons transfected with the Raichu-Rac biosensor. (A) Examples of Raichu-Rac-transfected neurons. (B) Evidence for validity of the assay using NGF (100 ng/ml) as a positive control for Rac1 activation as compared to vehicle control in developing primary cortical neurons derived from wild-type and CB1-deficient mouse embryos. (C) Time course and nature of bidirectional modulation of Rac1 activity by treatment with ACEA or AM-251 in comparison with vehicle. (D) Time course and nature of modulation of Rac1 activity based on the FRET measurements on the somatic areas. In panels B–D, FRET ratio in axonal growth cone at any given time was normalized to the averaged basal value within the same area prior to treatment. (E) Immunoblot analyses showing no changes in WAVE1 band density upon treatment with ACEA (100 nM) or AM251 (600 nM) as compared to vehicle treatment in cortical neurons derived from wild-type mouse embryos and quantitative summary of cannabinoid-induced modulation of pSer397 WAVE1 levels normalized to total WAVE1 (*n* = 6–7 independent culture experiments). All graphs represent mean values ± SEM *p < 0.05 as compared to basal values within the group and †*p* < 0.05 as compared to the corresponding agonist values, one-way ANOVA followed by posthoc Tukey’s test.
(TIF)

S4 Fig siRNA-mediated WAVE1 knockdown using an independent WAVE1 siRNA that target different sequences shows similar phenotype. (A & B) Immunoblot representation of WAVE1-down-regulation upon siRNA delivery in developing cortical neurons (A) and the corresponding quantification (B) (*n* = 8). (C) Analysis on the F-actin dynamics based on cannabinoid-induced changes in LifeAct-GFP intensity (left graph) and area (right graph) over time in growth cones shows no changes in cultured cortical neurons with siRNA-mediated knockdown of WAVE1 (*n* = 6–7 neurons per group from three independent culture experiments). All graphs represent mean values ± SEM *p < 0.05, one-way ANOVA followed by posthoc Tukey’s test.
(TIF)

S5 Fig Analysis of F-actin activity in dendritic spines of mature cultured cortical neurons based on FRAP experiments. (A & B) Summary of FRAP values as a function of time expressed as one-phase exponential function curves fitted to the respective datasets from neurons treated with ACEA or vehicle from mature cultured cortical neurons that were treated with scrambled siRNA (A) or derived from CB1-deficient embryos (12–15 dendritic spines/group from four independent culture experiments). All graphs represent mean values ± SEM.
(TIF)

S6 Fig Analysis of WAVE1 activity based on western blot band densitometry in dorsal spinal horn of L3–L5 spinal segments following CFA. (A) Immunoblot analyses showing no changes in total WAVE1 band density 24 h post CFA treatment. (B) Quantitative summary of CFA-induced reduced levels of pSER397 WAVE1 levels normalized to total WAVE1, which is partially and significantly reversed by intrathecal ACEA as compared to vehicle (*n* = 5 mice/group). All graphs represent mean values ± SEM. *p < 0.05 as compared to basal values within the group and †*p* < 0.05 as compared to corresponding values in the vehicle group, one-way ANOVA followed by posthoc Tukey’s test.
(TIF)

S1 Table List of potential CB1 interactors identified in protemic-MS analyses, ranked based on their rPQ score. (PDF)

## Abbreviations

2-AG: 2-arachidonylglycerol
ABI1/2: Abelson-interacting protein ½
ARP2/3: actin related protein 2/3
ACEA: arachidonyl-2’-chloroethylamide
CAMKII: Ca^2+^/calmodulin-dependent protein kinase II
CB1: cannabinoid type 1 receptor
CB2: cannabinoid type 2 receptor
CDK5: cyclin-dependent kinase 5
CFA: complete Freund’s adjuvant
CNS: central nervous system
COS: CV-1 in Origin with SV40 genes
CYFIP2: cytoplasmic FMR1 interacting protein 2
DIV: days in vitro
EGFP: enhanced green fluorescent protein
F-actin: filamentous actin
FRAP: fluorescence recovery after photobleaching
FRET: fluorescence resonance energy transfer
G-actin: actin monomers
GPCR: G-protein coupled receptor
LTP: long-term potentiation
MS: mass spectrometry
NCKAP1: NCK-associated protein 1
NGF: nerve growth factor
PBS: phosphate-buffered saline
pGAP43: phosphorylated growth-associated protein 43
PTX: pertussis toxin
rAAV: recombinant adeno-associated virions
rAAV-CamKII-GFP: rAAV vector expressing GFP under the promoter elements of the mouse CaMKII gene
rAAV-EGFP-CB1: recombinant adeno-associated virions expressing enhanced green fluorescent protein-tagged CB1
rPQ: relative peptide query
WAVE1: Wiskott-Aldrich syndrome protein-family verprolin-homologous protein 1
WRC: WAVE regulatory complex
