# Supplementary figures and images for: The Cannabinoid Receptor CB1 Interacts with the WAVE1 Complex and Plays a Role in Actin Dynamics and Structural Plasticity in Neurons
Source: PLoS Biol. 2015 Oct 23;13(10):e1002286. doi: 10.1371/journal.pbio.1002286 (PMC4619884; doi:10.1371/journal.pbio.1002286)

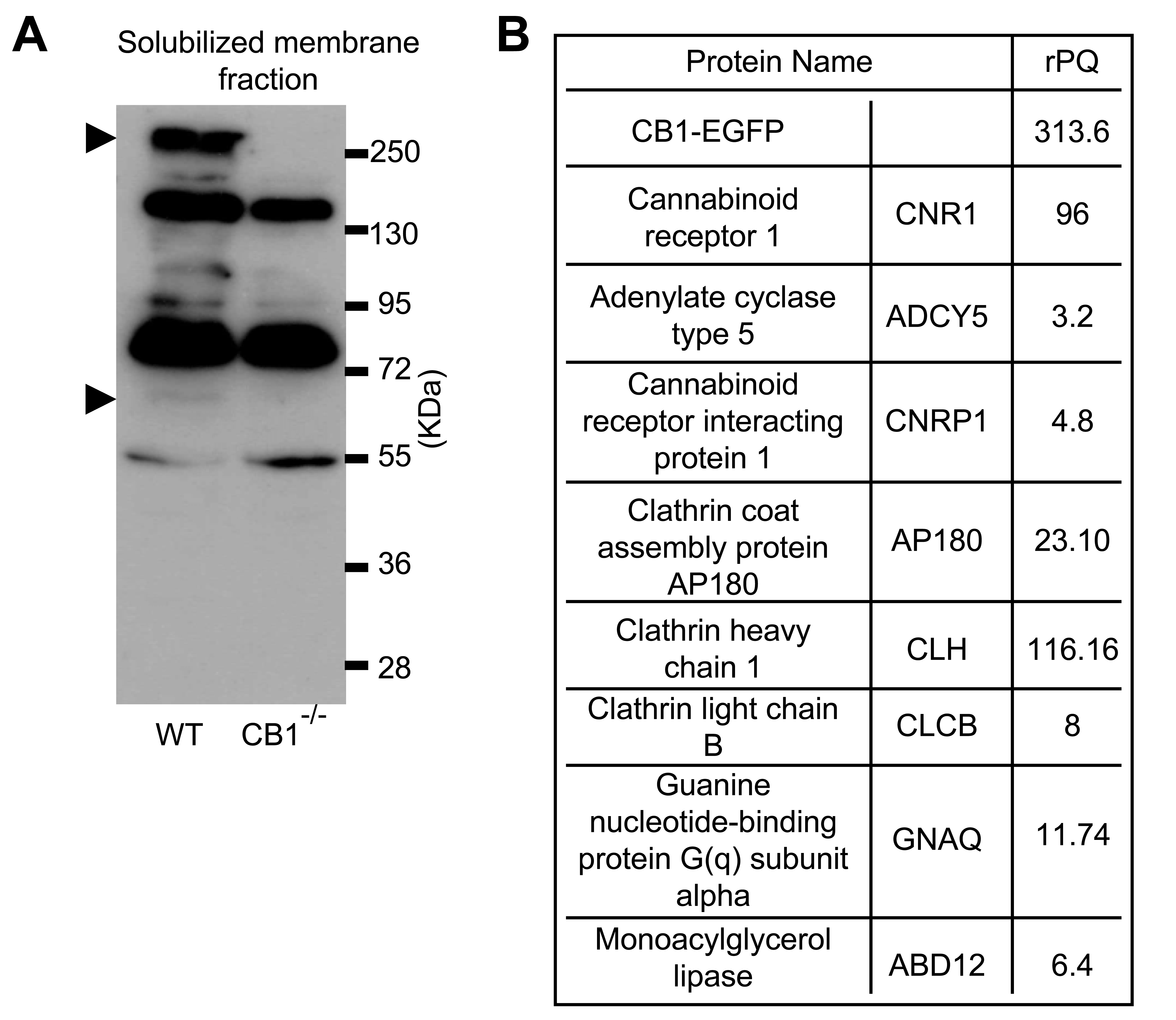

Supplement: S1 Fig — (A) Validation of bands recognized by the anti-CB1 antibody in membrane fractions derived from wild-type mice (WT) and mice lacking CB1 globally (CB1-/- mice). Only the bands indicated by black arrowheads, constituting monomeric and multimeric CB1, are specific and the uppermost band corresponds to the multimeric CB1-EGFP band indicated by arrow and arrowhead in panel A. (B) Overview of known (published) interaction partners of CB1 found via LC-MS analysis on immunoprecipitates derived from mouse cortically expressing CB1-EGFP, but not in mice expressing EGFP alone. Shown are rPQ, with values above four indicating significant interactions. (TIF) [file pbio.1002286.s002.tif]

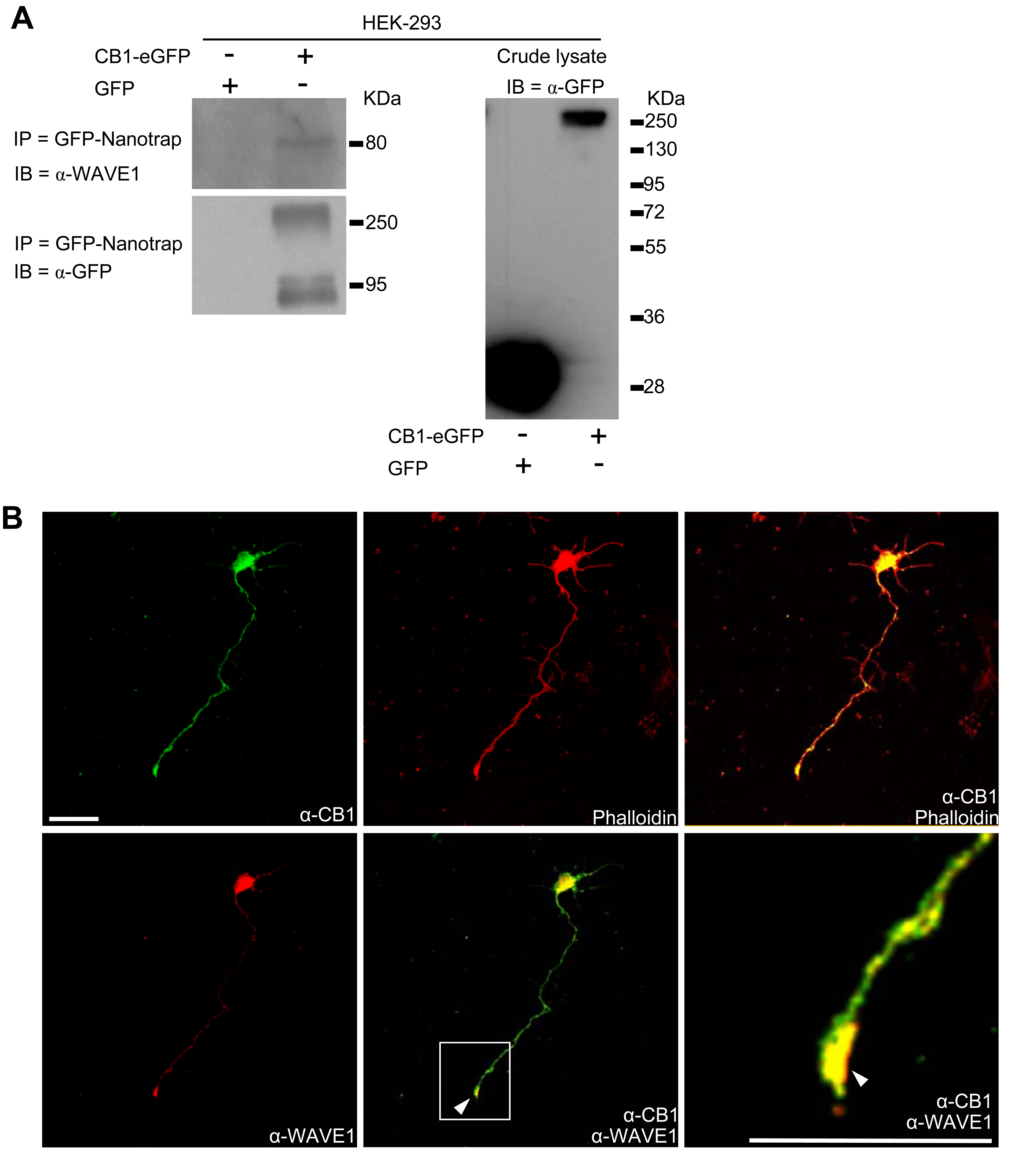

Supplement: S2 Fig — (A) In HEK cells transfected heterologously with CB1-EGFP or with GFP, endogenous WAVE1 coimmunoprecipitated with CB-EGFP, but not with GFP alone. Expression controls for GFP and CB1-EGFP in transfected cells are shown on the right. (B) Pseudocolored images showing colocalization between endogenously expressed CB1, actin, and WAVE1 in immunostained cultured cortical neurons. Magnified image represent white box inset. White arrowheads in inset and magnified image point to the growth cone. Scale bars represent 20 μm. (TIF) [file pbio.1002286.s003.tif]

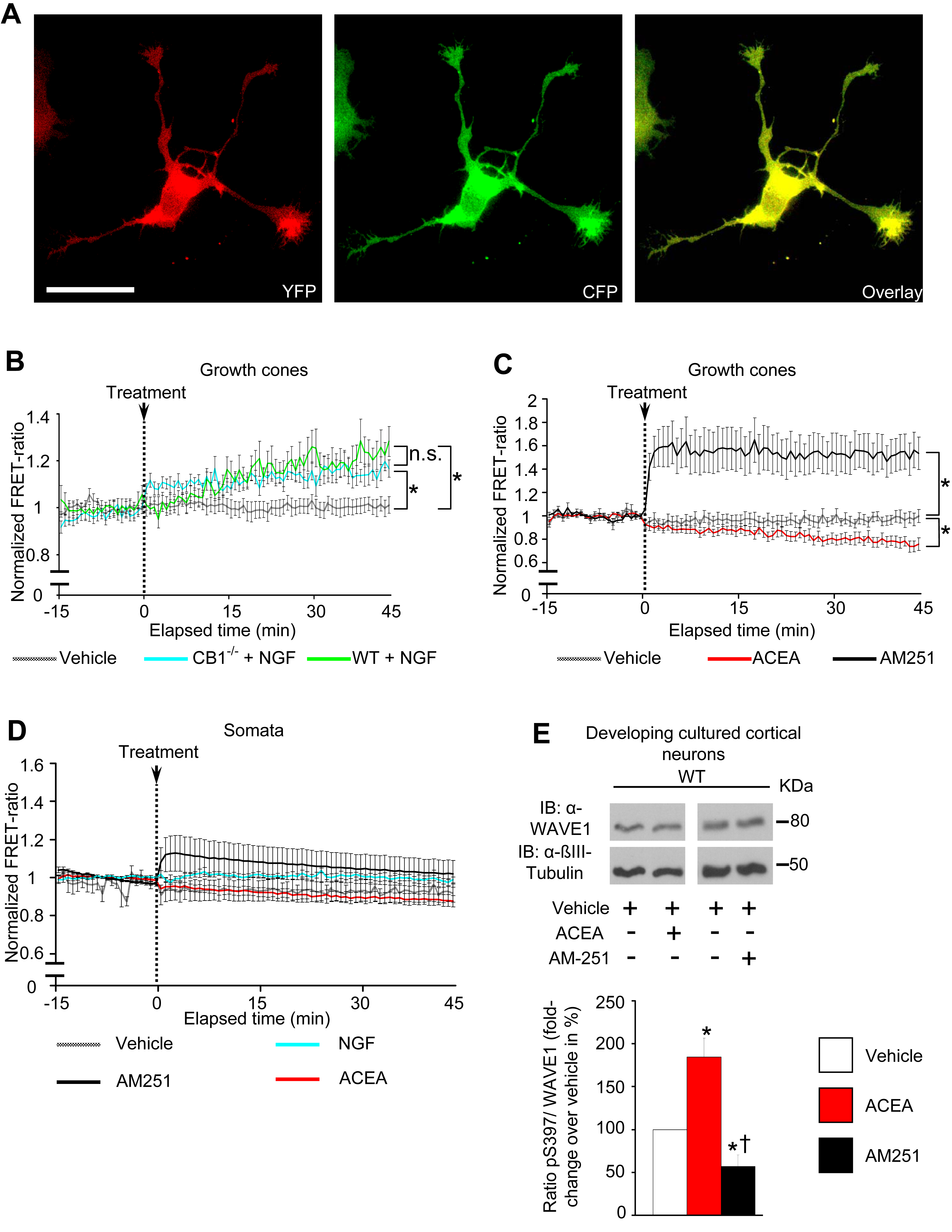

Supplement: S3 Fig — (A) Examples of Raichu-Rac-transfected neurons. (B) Evidence for validity of the assay using NGF (100 ng/ml) as a positive control for Rac1 activation as compared to vehicle control in developing primary cortical neurons derived from wild-type and CB1-deficient mouse embryos. (C) Time course and nature of bidirectional modulation of Rac1 activity by treatment with ACEA or AM-251 in comparison with vehicle. (D) Time course and nature of modulation of Rac1 activity based on the FRET measurements on the somatic areas. In panels B–D, FRET ratio in axonal growth cone at any given time was normalized to the averaged basal value within the same area prior to treatment. (E) Immunoblot analyses showing no changes in WAVE1 band density upon treatment with ACEA (100 nM) or AM251 (600 nM) as compared to vehicle treatment in cortical neurons derived from wild-type mouse embryos and quantitative summary of cannabinoid-induced modulation of pSer397 WAVE1 levels normalized to total WAVE1 (n = 6–7 independent culture experiments). All graphs represent mean values ± SEM *p < 0.05 as compared to basal values within the group and †p < 0.05 as compared to the corresponding agonist values, one-way ANOVA followed by posthoc Tukey’s test. (TIF) [file pbio.1002286.s004.tif]

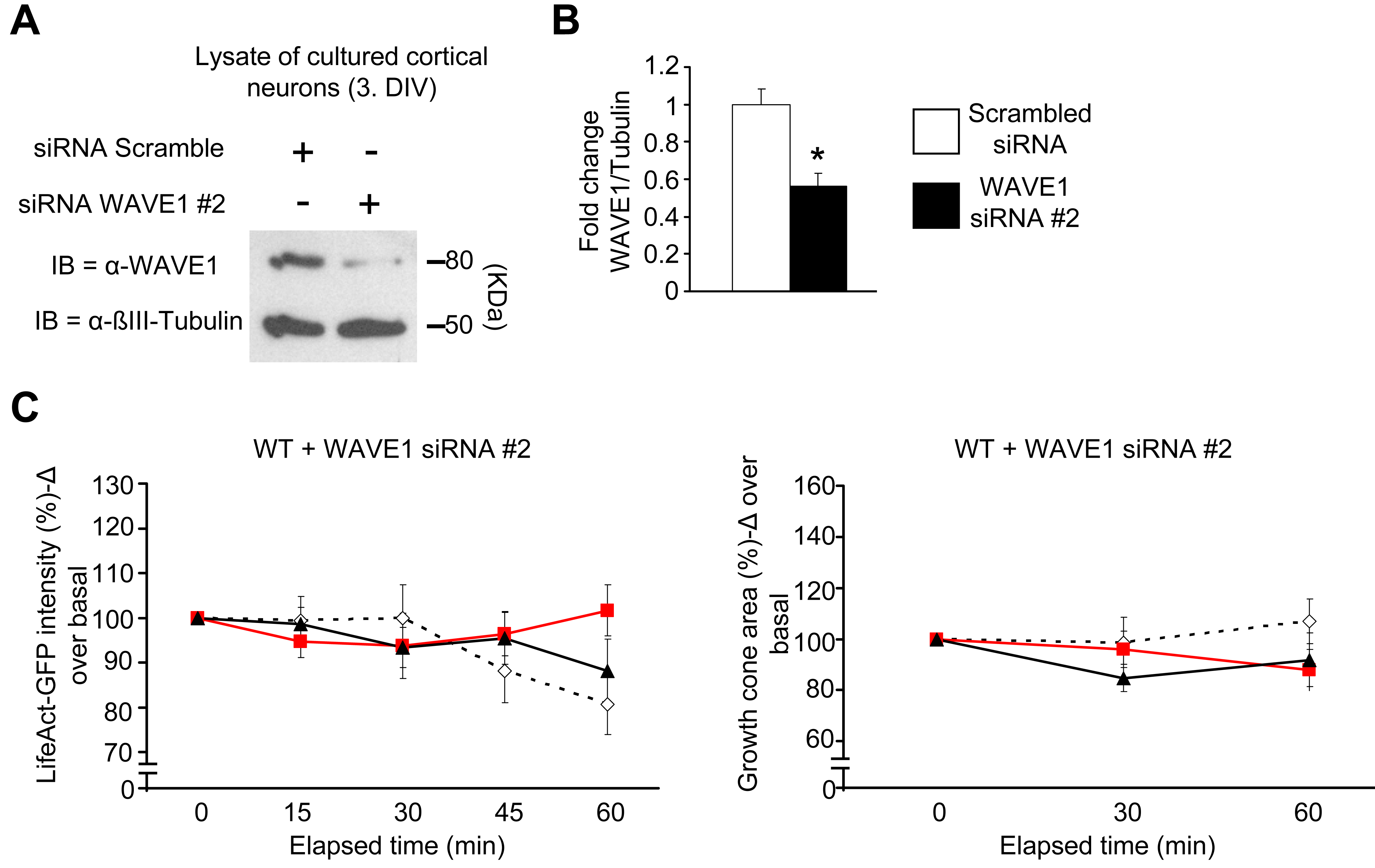

Supplement: S4 Fig — (A & B) Immunoblot representation of WAVE1-down-regulation upon siRNA delivery in developing cortical neurons (A) and the corresponding quantification (B) (n = 8). (C) Analysis on the F-actin dynamics based on cannabinoid-induced changes in LifeAct-GFP intensity (left graph) and area (right graph) over time in growth cones shows no changes in cultured cortical neurons with siRNA-mediated knockdown of WAVE1 (n = 6–7 neurons per group from three independent culture experiments). All graphs represent mean values ± SEM *p < 0.05, one-way ANOVA followed by posthoc Tukey’s test. (TIF) [file pbio.1002286.s005.tif]

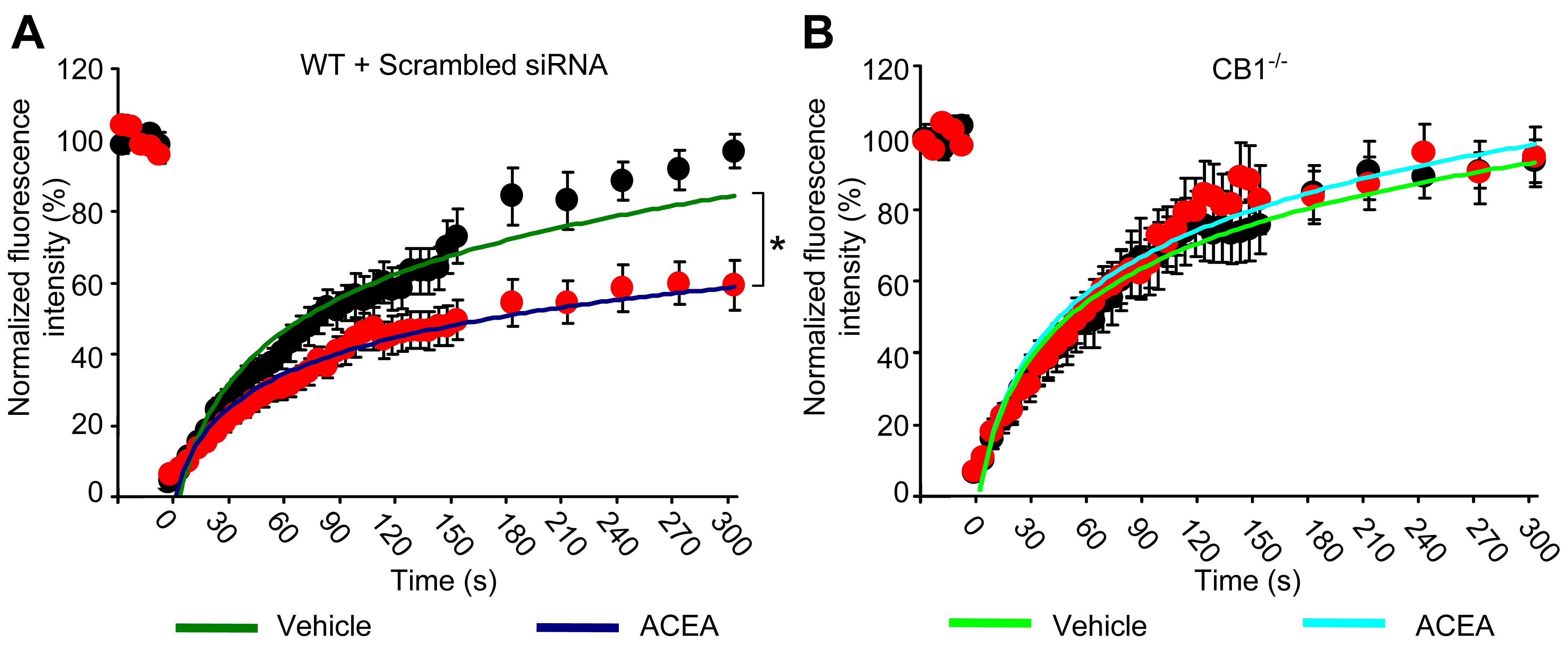

Supplement: S5 Fig — (A & B) Summary of FRAP values as a function of time expressed as one-phase exponential function curves fitted to the respective datasets from neurons treated with ACEA or vehicle from mature cultured cortical neurons that were treated with scrambled siRNA (A) or derived from CB1-deficient embryos (12–15 dendritic spines/group from four independent culture experiments). All graphs represent mean values ± SEM. (TIF) [file pbio.1002286.s006.tif]

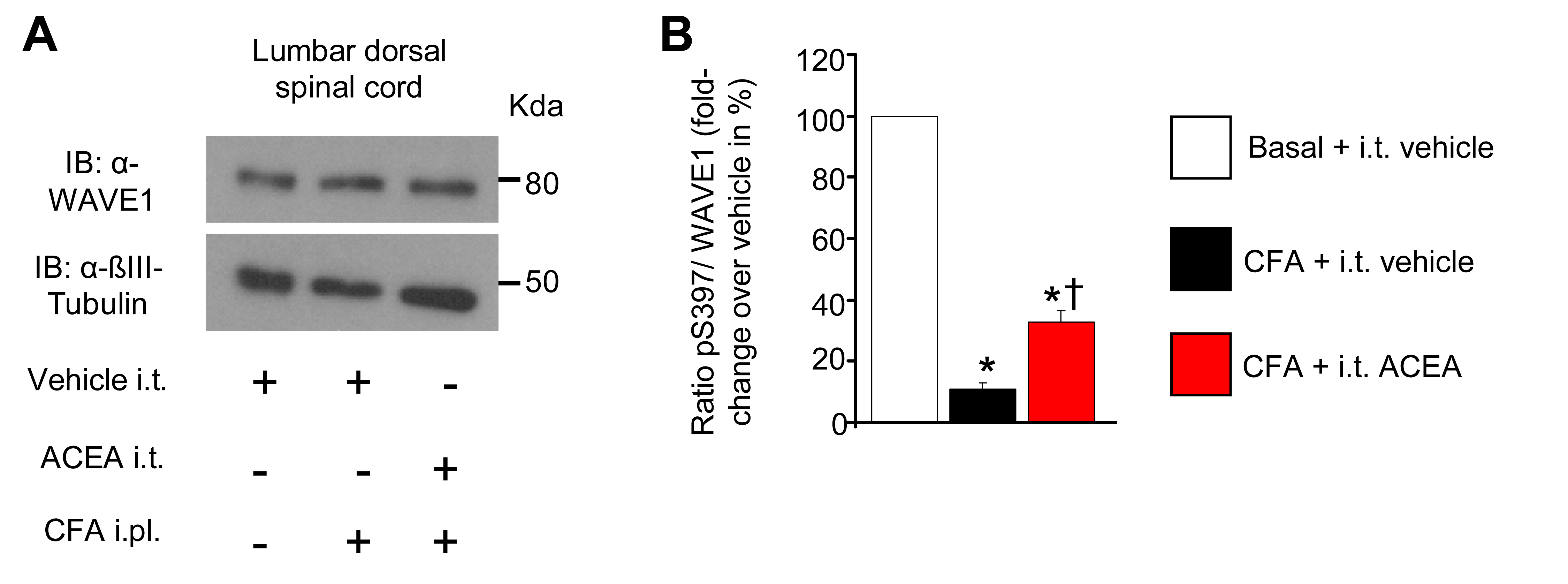

Supplement: S6 Fig — (A) Immunoblot analyses showing no changes in total WAVE1 band density 24 h post CFA treatment. (B) Quantitative summary of CFA-induced reduced levels of pSER397 WAVE1 levels normalized to total WAVE1, which is partially and significantly reversed by intrathecal ACEA as compared to vehicle (n = 5 mice/group). All graphs represent mean values ± SEM. *p < 0.05 as compared to basal values within the group and †p < 0.05 as compared to corresponding values in the vehicle group, one-way ANOVA followed by posthoc Tukey’s test. (TIF) [file pbio.1002286.s007.tif]
