# Supplementary material for: The Cannabinoid Receptor CB1 Interacts with the WAVE1 Complex and Plays a Role in Actin Dynamics and Structural Plasticity in Neurons
Source: PLoS Biol. 2015 Oct 23;13(10):e1002286. doi: 10.1371/journal.pbio.1002286 (PMC4619884; doi:10.1371/journal.pbio.1002286)
Supplement: S1 Table — (PDF) [file pbio.1002286.s008.pdf]

Rank	Protein Name		rPQ Score
1	CB1-eGFP		313.60
2	Clathrin heavy chain 1	CLH	116.16
3	Cannabinoid receptor 1	CNR1	96.00
4	Sodium/potassium-transporting ATPase subunit alpha-2	AT1A2	85.53
5	AP-2 complex subunit alpha-2	AP2A2	80.57
6	AP2-associated protein kinase 1	AAK1	77.29
7	Wiskott-Aldrich syndrome protein family 1	WASF1	48.20
8	Nck-associated protein 1	NCKP1	45.01
9	Dynamin-1	DYN1	41.80
10	Amphiphysin	AMPH	38.95
11	Synaptic vesicle glycoprotein 2A	SV2A	36.06
12	Band 4.1-like protein 3	E41L3	35.59
13	Septin-9	SEPT9	35.41
14	Cytochrome b-c1 complex subunit Rieske, mitochondrial	UCRI	34.35
15	V-type proton ATPase 116 kDa subunit a isoform 1 OS=Mus musculus GN=Atp6v0a1 PE=1 SV=2	VPP1	33.99
16	Adenylyl cyclase-associated protein 1	CAP1	33.78
17	Catenin delta-2	CTND2	32.43
18	Cytoplasmic FMR1-interacting protein 2	CYFP2	32.22
19	Voltage-dependent anion-selective channel protein 3	VDAC3	32.00
20	Glial fibrillary acidic protein	GFAP	31.15
21	cAMP-dependent protein kinase type II-beta regulatory subunit	KAP3	30.40
22	Sodium-driven chloride bicarbonate exchanger	S4A10	30.40
23	Vacuolar protein sorting-associated protein 35	VPS35	30.40
24	Plasma membrane calcium-transporting ATPase 2	AT2B2	29.81
25	Plasma membrane calcium-transporting ATPase 2	AT2B2	29.81
26	Ankyrin-2	ANK2	28.84
27	V-type proton ATPase subunit H	VATH	28.80
28	Cystein & glycine rich protein 1	CSRP1	27.45
29	Microsomal glutathione S-transferase 3	MGST3	25.60
30	Solute carrier family 12 member 5	S12A5	24.76

Rank	Protein Name		rPQ Score
31	Contactin-1	CNTN1	24.71
32	Clathrin coat assembly protein AP180	AP180	23.11
33	Band 4.1-like protein 2	E41L2	22.47
34	Ras-related protein Rab-3C	RAB3C	22.40
35	Immunoglobulin superfamily member 8	IGSF8	21.81
36	Homer protein homolog 1	HOME1	19.49
37	Na(+)/H(+) exchange regulatory cofactor NHE-RF1	NHRF1	19.20
38	Neurofilament light polypeptide	NFL	18.73
39	Guanine nucleotide-binding protein G(z) subunit alpha	GNAZ	17.74
40	V-type proton ATPase subunit B, brain isoform	VATB2	17.18
41	Synaptic vesicle glycoprotein 2B	SV2B	16.79
42	Electrogenic sodium bicarbonate cotransporter 1	S4A4	16.44
43	Tenascin-R	TENR	16.33
44	Major prion protein	PRIO	16.27
45	Septin-4	SEPT4	16.19
46	Brain-specific angiogenesis inhibitor 1-associated protein 2	BAIP2	16.00
47	Spectrin beta chain, erythrocyte	SPTB1	16.00
48	CaM kinase-like vesicle-associated protein	CAMKV	15.03
49	Rabphilin-3A	RP3A	14.63
50	DnaJ homolog subfamily A member 2	DNJA2	14.60
51	cAMP-dependent protein kinase catalytic subunit alpha	KAPCA	14.40
52	PH and SEC7 domain-containing protein 3	PSD3	13.46
53	Neural cell adhesion molecule 1	NCAM1	13.45
54	Coiled-coil-helix-coiled-coil-helix domain-containing protein 6	CHCH6	13.20
55	G protein-regulated inducer of neurite outgrowth 1	GRIN1	13.08
56	Abl interactor 2	ABL2	12.80
57	Gap junction alpha-1 protein	CXA1	12.80
58	Junction plakoglobin	PLAK	12.80
59	Ras-related protein Rab-35	RAB35	12.80
60	V-type proton ATPase subunit d 1	VA0D1	12.00

Rank	Protein Name		rPQ Score
61	Elongation factor 1-alpha 1	EF1A1	11.91
62	Guanine nucleotide-binding protein G(q) subunit alpha	GNAQ	11.74
63	Gephyrin	GEPH	11.45
64	Lipid phosphate phosphohydrolase 3	LPP3	11.20
65	Ras-related protein Rab-5C	RAB5C	11.20
66	Secretory carrier-associated membrane protein 1	SCAM1	11.20
67	V-type proton ATPase catalytic subunit A	VATA	10.92
68	Spectrin beta chain, brain 1	SPTB2	10.91
69	LIM and SH3 domain protein 1	LASP1	10.30
70	Hepatocyte cell adhesion molecule	HECAM	9.95
71	Neurotrimin	NTRI	9.80
72	Contactin-associated protein 1	CNTP1	9.68
73	cAMP-dependent protein kinase type II-alpha regulatory subunit	KAP2	9.60
74	Dual specificity mitogen-activated protein kinase kinase 1	MP2K1	9.60
75	F-actin-capping protein subunit alpha-2	CAZA2	9.60
76	Lymphocyte antigen 6H	LY6H	9.60
77	Voltage-dependent L-type calcium channel subunit beta-4	CACB4	9.60
78	V-type proton ATPase subunit C 1	VATC1	9.60
79	Spectrin alpha chain, brain	SPTA2	9.19
80	α-enolase	ENOA	9.06
81	Neurofilament medium polypeptide	NFM	9.00
82	Ras-related C3 botulinum toxin substrate 1	RAC1	8.98
83	Myelin-associated glycoprotein	MAG	8.60
84	Leukocyte surface antigen CD47	CD47	8.33
85	Ras-related protein Rab-11A	RB11A	8.30
86	Regulator of G-protein signaling 7	RGS7	8.17
87	Clathrin light chain B	CLCB	8.00
88	Myristoylated alanine-rich C-kinase substrate	MARCS	8.00
89	WD repeat-containing protein 7	WDR7	8.00
90	Sodium/potassium-transporting ATPase subunit beta-2	AT1B2	7.53

Rank	Protein Name		rPQ Score
91	4F2 cell-surface antigen heavy chain	4F2	7.05
92	Cell adhesion molecule 3	CADM3	6.87
93	Beta-soluble NSF attachment protein	SNAB	6.83
94	Voltage-dependent anion-selective channel protein 2	VDAC2	6.81
95	Dynamin-1-like protein	DNM1L	6.60
96	Vesicle-associated membrane protein-associated protein B	VAPB	6.56
97	Actin-related protein 2/3 complex subunit	ARPC5	6.40
98	Cystein-rich protein 2	CRIP2	6.40
99	Endophilin-A1	SH3G2	6.40
100	Endothelin B receptor-like protein 2	ETBR2	6.40
101	Monoacylglycerol lipase ABHD12	ABD12	6.40
102	Phosphatidylinositol 4,5-bisphosphate 5-phosphatase A	PI5PA	6.40
103	Plexin-A1	PLXA1	6.40
104	Transmembrane protein 65	TMM65	6.40
105	Coronin-1A	COR1A	5.39
106	V-type proton ATPase subunit E 1	VATE1	5.36
107	PKC and casein kinase substrate in neurons protein	PACN1	5.27
108	GTPase HRas	RASH	5.10
109	Catenin beta-1	CTNB1	4.80
110	CB1 cannabinoid receptor-interacting protein 1	CNRP1	4.80
111	Dolichyl-diphosphooligosaccharide--protein glycosyltransferase 48 kDa subunit	OST48	4.80
112	Flotillin-1	FLOT1	4.80
113	Protein bassoon	BSN	4.80
114	Rho-related GTP-binding protein RhoG	RHOG	4.80
115	Syntaxin-12	STX12	4.80
116	Transforming protein RhoA	RHOA	4.80
117	Transgelin-3	TAGL3	4.80
118	Voltage-gated potassium channel subunit beta-2	KCAB2	4.26
119	Sodium- and chloride-dependent GABA transporter 3	S6A11	4.07
120	Sodium- and chloride-dependent GABA transporter 1	SC6A1	3.83

Rank	Protein Name		rPQ Score
121	Vesicular inhibitory amino acid transporter	VIAAT	3.73
122	DnaJ homolog subfamily C member 5	DNJC5	3.60
123	Disintegrin and metalloproteinase domain-containing protein 23	ADA23	3.50
124	Tyrosine-protein phosphatase non-receptor type substrate 1	SHPS1	3.47
125	Catenin alpha-2	CTNA2	3.35
126	Protein CDV3 (carnitine deficiency-associated gene expressed in ventricle)	CDV3	3.32
127	Adenylate cyclase type 5	ADCY5	3.20
128	Dematin	DEMA	3.20
129	DmX-like protein 2	DMXL2	3.20
130	Dolichyl-diphosphooligosaccharide--protein glycosyltransferase subunit 2	RPN2	3.20
131	F-actin-capping protein subunit beta	CAPZB	3.20
132	Gamma-aminobutyric acid receptor subunit beta-2	GBRB2	3.20
133	Neural cell adhesion molecule 2	NCAM2	3.20
134	Solute carrier family 2, facilitated glucose transporter member 1	GTR1	3.20
135	Talin-2	TLN-2	3.20
136	Disks large homolog 3	DLG3	2.53
137	AP-2 complex subunit alpha-1	AP2A1	2.11
138	Veiscle-fusing ATPase	NSF	1.96
139	Excitatory amino acid transporter 2	EAA2	1.88
140	AP-2 complex subunit beta	AP2B1	1.81
141	Synapsin 1	SYN1	1.63
142	Septin-5	SEPT5	1.52
143	AP-2 complex subunit mu	AP2M1	1.45
144	Sodium/potassium-transporting ATPase subunit alpha-1	AT1A1	1.42
145	2', 3'-cyclic-nucleotide 3'-phosphodiesterase	CN37	1.38
146	Septin-2	SEPT2	1.33
147	Septin-7	SEPT7	1.29
148	Guanin nucleotide binding protein/ Goα	GNAO	1.28
149	Septin 11	SEP11	1.18
150	Septin-6	SEPT6	1.17
